# Cardiomyocyte Membrane Structure and cAMP Compartmentation Produce Anatomical Variation in β_2_AR-cAMP Responsiveness in Murine Hearts

**DOI:** 10.1016/j.celrep.2018.03.053

**Published:** 2018-04-10

**Authors:** Peter T. Wright, Navneet K. Bhogal, Ivan Diakonov, Laura M.K. Pannell, Ruwan K. Perera, Nadja I. Bork, Sophie Schobesberger, Carla Lucarelli, Giuseppe Faggian, Anita Alvarez-Laviada, Manuela Zaccolo, Timothy J. Kamp, Ravi C. Balijepalli, Alexander R. Lyon, Sian E. Harding, Viacheslav O. Nikolaev, Julia Gorelik

**Affiliations:** 1 Myocardial Function, National Heart and Lung Institute, Imperial College London, ICTEM, Hammersmith Hospital, Du Cane Road, London W12 0NN, UK; 2 Institute of Experimental Cardiovascular Research, University Medical Center Hamburg-Eppendorf, 20246 Hamburg, Germany; 3 Department of Cardiac Surgery, University of Verona School of Medicine, Azienda Ospedalieria Universitaria Integrata, Borgo Trento Piazzale A. Stefani, 37126 Verona, Italy; 4 Department of Physiology, Anatomy and Genetics, University of Oxford, Oxford OX1 3QX, UK; 5 Department of Medicine, University of Wisconsin Madison, 1111 Highland Ave., Madison, WI 53705-2275, USA; 6 NIHR Cardiovascular Biomedical Research Unit, Royal Brompton Hospital, London SW7 3AZ, UK

**Keywords:** cAMP, microdomains, cardiomyocytes, T-tubules, caveolae, β_2_ adrenoceptor

## Abstract

Cardiomyocytes from the apex but not the base of the heart increase their contractility in response to β_2_-adrenoceptor (β_2_AR) stimulation, which may underlie the development of Takotsubo cardiomyopathy. However, both cell types produce comparable cytosolic amounts of the second messenger cAMP. We investigated this discrepancy using nanoscale imaging techniques and found that, structurally, basal cardiomyocytes have more organized membranes (higher T-tubular and caveolar densities). Local membrane microdomain responses measured in isolated basal cardiomyocytes or in whole hearts revealed significantly smaller and more short-lived β_2_AR/cAMP signals. Inhibition of PDE4, caveolar disruption by removing cholesterol or genetic deletion of Cav3 eliminated differences in local cAMP production and equilibrated the contractile response to β_2_AR. We conclude that basal cells possess tighter control of cAMP because of a higher degree of signaling microdomain organization. This provides varying levels of nanostructural control for cAMP-mediated functional effects that orchestrate macroscopic, regional physiological differences within the heart.

## Highlights

• Cardiomyocyte membrane organization varies in degree between regions of the heart
• Differences in structural organization affect adrenergic signaling via β_2_AR
• Reduced organization allows β_2_AR-cAMP to influence contractility in myocardial apex
• Variability in cell structure may allow differential response of heart regions

## Graphical Abstract

**Figure:**
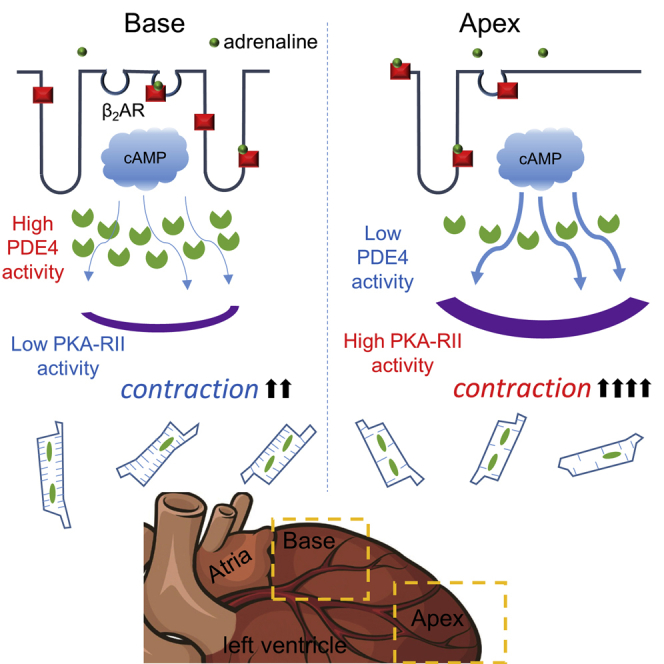

## Teaser

Wright et al. present evidence that cardiomyocyte membrane organization (T-tubule regularity and caveolar number) varies between myocardial regions. The reduced membrane organization of cells from the myocardial apex allows β_2_AR-cAMP to influence PKA_RII domains. As a result, β_2_AR stimulation enhances apically but not basally derived cardiomyocyte contractility.

## Introduction

The mammalian heart is a complex 3D structure. The ventricular myocardium is composed of myofibrils, which form layers (Lunkenheimer et al., 2006). Previous research has generally investigated electrophysiological differences between the epicardial and endocardial layers of the ventricles (Stankovicova et al., 2000, Szentadrassy et al., 2005). There is a limited understanding of regional differences, longitudinally, between the apical and basal myocardium. Historically, research into G-protein-coupled receptor (GPCR) function in isolated cardiomyocytes has not accounted for the potential heterogeneity of cardiomyocytes in healthy tissue. Cardiac beta-adrenoceptor (βAR) density is relatively increased in the basal myocardium of dogs (Kawano et al., 2003). A differential “apico-basal” gradient of sympathetic innervation is observed in human samples (Mori et al., 1993). The β-adrenergic responsiveness of the apical myocardium is higher than the basal in rat (Heather et al., 2009), feline (Lathers et al., 1986), and rabbit hearts (Mantravadi et al., 2007). Enhancement of sympathetic input into specific cardiac regions, although possibly supporting efficient cardiac function, may make the apex vulnerable to toxic catecholamine levels during severe stress.

Infusion of the non-specific βAR agonist isoproterenol (ISO) is a classical method of inducing infarct-like damage and chronic heart failure in small animal models. This selectively damages the apex (Rona et al., 1959). In recent years, Takotsubo syndrome (TTS), has been described (Lyon et al., 2016). Multiple preclinical models of TTS, which predominantly afflicts the apex, have been published, involving similar methodologies (Paur et al., 2012, Shao et al., 2013a, Shao et al., 2013b). Previously, we discovered that isolated cardiomyocytes from the apex of rat hearts increased their fractional shortening to a much greater degree than those isolated from the basal segment following β_2_AR stimulation (Paur et al., 2012). However, real-time measurements of cytoplasmic β_2_AR-cyclic AMP (cAMP) responses were not different between apical and basal cells. This suggested that differences in regional contractile response were not simply the result of an increased density of β_2_AR in the apical cardiomyocytes. In this paper, we investigate the role of cAMP control and compartmentation in this phenomenon.

To control contractility in cardiomyocytes, cAMP must engage a protein kinase A (PKA)-dependent (Bers, 2002) signaling pathway. Because multiple GPCR-based pathways acting via G_s_ exist in cardiomyocytes, different pools of PKA bound to different A kinase anchoring proteins (AKAPs) are present (Di Benedetto et al., 2008). This allows selective, specific activation of different cell effector systems by different pools of PKA (Carr et al., 1992). The protein kinase A regulatory subunit 2 (PKA_RII) domain controls cardiomyocyte contractility. In this paper, we make use of a fluorescence resonance energy transfer (FRET)-based cAMP sensor that includes a segment of the RII regulatory peptide, allowing it to be localized with membrane-bound PKA and, thus, measure cellular cAMP responses in this cellular region. We also probe the membrane-based control of cAMP by using a plasma membrane-localized FRET sensor (pmEpac2). The cardiomyocyte membrane provides the structural basis for information processing via GPCRs (Di Benedetto et al., 2008, Buxton and Brunton, 1983). The coherence and specificity of βAR signaling is produced by the rapid inactivation or degradation of secondary messengers (Yang et al., 2014, Nikolaev et al., 2006a) as well as removal of the receptor (Liu et al., 2012) and dephosphorylation of PKA targets (Macdougall et al., 2012). Elements of the cardiomyocyte plasma membrane are involved in receptor recycling and provide a mechanism by which phosphodiesterases (PDEs) are made more efficient (Willoughby et al., 2006). The transverse (t)-tubular system and caveolae are the most well characterized organizational elements within the cardiomyocyte membrane with respect to signal transduction (Nikolaev et al., 2010, Wright et al., 2014, Razani et al., 2002, Head et al., 2005). β_2_ARs have been shown to be localized within caveolae in cardiac tissue and cells. Removal of β_2_ARs from caveolae via removal of cholesterol or caveolin-3 (Cav3) results in enhancement of β_2_AR-mediated contractile responses (Macdougall et al., 2012).

In this paper, we demonstrate that apical cardiomyocytes from rat and mouse hearts exhibit greater contractile responses following β_2_AR stimulation than those from the base. We show regional differences in membrane organization and a subsequent increase in the stringency of the control of local cAMP microdomains in basal cardiomyocytes by Cav3-associated PDE subtype 4 activity. This has the potential to explain the increased sensitivity of the apical myocardium to circulating catecholamines in pathology and is an example of nanostructural changes in cells leading to differential regional effects on function within organs.

## Results

### Rat Apical Cardiomyocytes Display Increased Responsiveness to β_2_AR Stimulation Compared with Basal Cardiomyocytes from the Same Heart

**Figure 1:**
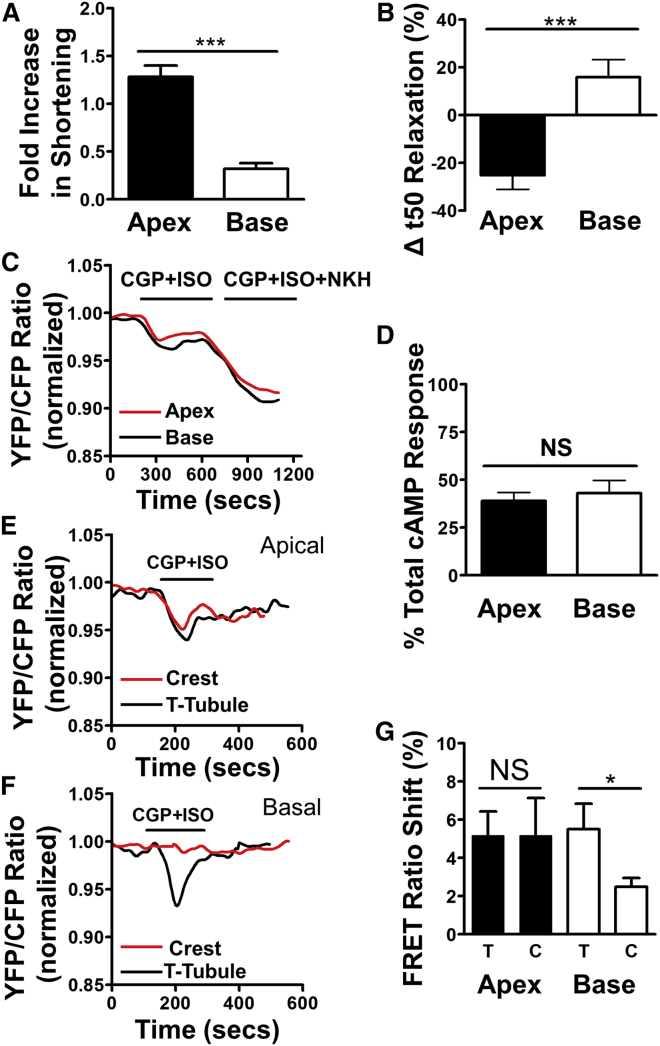
Contraction and cAMP Production in Response to β_2_AR Stimulation (Rat Apical and Basal Cells) (A) Cell shortening increases after β_2_AR stimulation (1 μM ISO + 300 nM CGP). N, n = (6) 11/9, p < 0.001, apex versus base, unpaired t test. (B) Relative change in speed of relaxation. N, n = (6) 11/9, p < 0.001, apex versus base, unpaired t test. (C) Representative traces showing the β_2_AR FRET response measured by the cytosolic cAMP sensor cEPAC2-camps in apical and basal cardiomyocytes. (D) Calculation of the amount of cAMP production after β_2_AR stimulation relative to absolute maximum of production after stimulation with NKH477. N, n = (10) 36/34, not statistically significant (NS), apex versus base, unpaired t test. (E and F) Example traces of FRET responses after local stimulation of T-tubules or crest regions in apical (E) or basal (F) cells. (G) Comparison of apical (tubule/crest, N, n = (9) 15/13, NS) and basal (tubule/crest, N, n = (10) 21/20, p < 0.05, unpaired t test) responses. Error bars indicate SEM. See also Figure S2.

We isolated cardiomyocytes from the apex and base of the left ventricle of the rat heart (Figure S1A); their gross morphology does not differ (Figure S1B). Contraction was measured under stimulation (0.5 Hz, 50 V). Apical cardiomyocytes display a greater increase in their shortening after β_2_AR stimulation than cells derived from the basal myocardium. Normalizing this increase to baseline as well as uncorrected fractional shortening data (Figure 1A and Figure S2A) reveal a significant difference between the responses of apical and basal cardiomyocytes. Apical cardiomyocytes also relax with higher velocity (Figure 1B), taking less time to reach 50% diastolic length after β_2_AR stimulation. We confirmed that CGP20712A (CGP)+ISO specifically stimulate β_2_AR using β_2_AR knockout mice (Figures S2D and S2G). We also stimulated apical and basal cells with ISO alone (Figures S2E and S2H) and with ICI118,551 (ICI)+ISO to specifically stimulate β_1_AR (Figures S2F and S2I). These treatments stimulated the contraction of both cell types to the same degree (Figures S2D–S2I).

### β_2_AR-Dependent cAMP Levels Are Equal in the Cytosol of Apical and Basal Cardiomyocytes from Rats

We studied β_2_AR-dependent cAMP production in cells isolated from the apical or basal myocardium of rats, which were transfected with cytosolic FRET sensor Epac2-based cytosolic cAMP sensor (cEPAC2-camps) (Nikolaev et al., 2006b). Similar levels of cAMP were found to be induced in the cytoplasm of apical and basal cells following β_2_AR stimulation (Figures 1C and 1D). The data in Figure 1D are expressed as a percentage of total cAMP production capacity following stimulation of adenylate cyclase activity (via G_s_ protein stimulation with the forskolin analog NKH477). No statistically significant differences were observed between β_2_AR-dependent cAMP levels in apical and basal myocytes.

### Stimulated β_2_AR-cAMP Responses Are Observed to Be Equal on the T-Tubule and Sarcolemmal Crests of Apical but Not Basal Rat Cardiomyocytes

We have previously demonstrated that, in rat ventricular myocytes, β_2_AR-dependent cAMP production is mostly localized to T-tubules, in contrast to β_1_AR-dependent cAMP production (Nikolaev et al., 2010). We sought to determine whether there is a difference in the micro-domain response of local β_2_AR stimulation within apical and basal myocytes. Initially, scans of cellular topography were acquired using scanning ion conductance microscopy (SICM). β_2_ARs were then stimulated in either T-tubule openings or sarcolemma crests via the local application of ISO through the SICM nanopipette in the presence of selective blockade of β_1_ARs by CGP in the bath solution. We then measured the relative production of cAMP with the cytosolic FRET sensor cEPAC2-camps in the cell cytosol. We found that, in apical myocytes, β_2_AR-dependent cAMP is produced at a similar level in T-tubular and crest regions. However, in basal cells, a higher level of β_2_AR-dependent cAMP was produced in T-tubules relative to cell crests (Figures 1E–1G).

### Basal Cardiomyocytes from Rats Exhibit a Greater Degree of Membrane Organization in Comparison with Apical Cells

**Figure 2:**
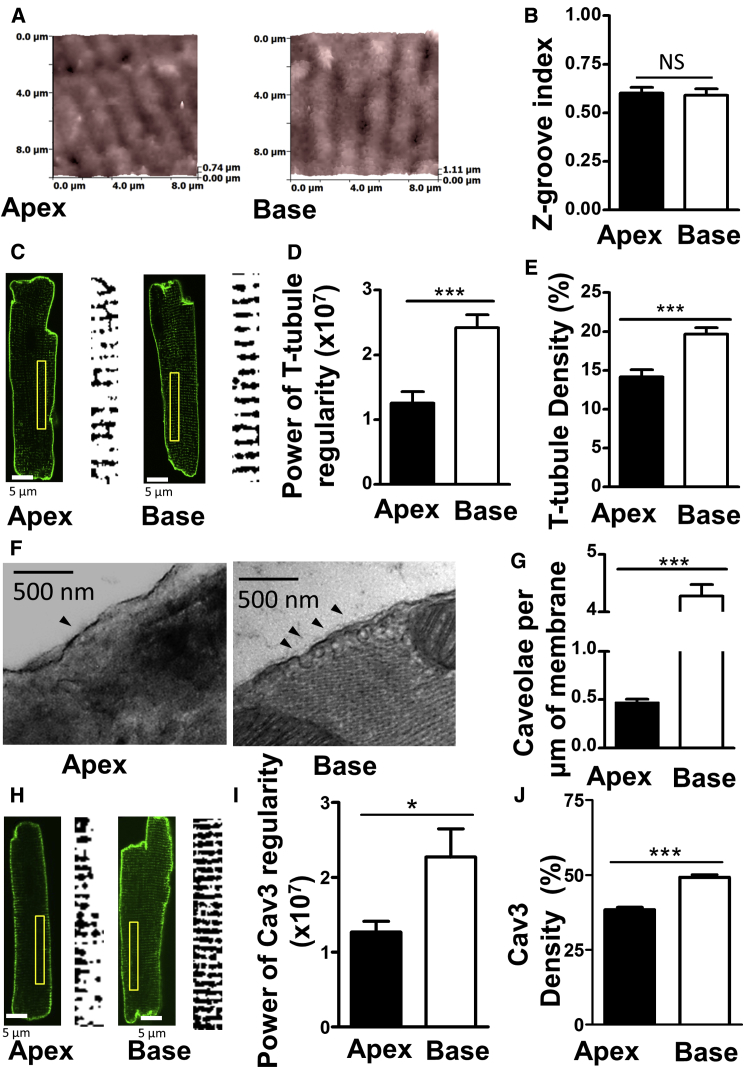
Intracellular Organization of Apical and Basal Myocytes from Rats (A) Representative SICM images of the surface topography of apical and basal cardiomyocytes. (B) Quantification of the degree of myocyte surface structural organization by Z-groove index (apical/basal, N, n = (5) 45/44, NS, unpaired t test). (C) Tubular membrane organization of di-8ANNEPPS-stained apical and basal cardiomyocytes. Insets show the areas chosen to make binary images for analysis. (D and E) Quantification of the relative regularity (D) and density (E) of T-tubules (N, n = (3) 15, p < 0.001). (F) Examples of electron micrographs showing plasma membrane cross-sections of apical and basal myocytes; N = 10. (G) Quantification of the density of caveolae in apical and basal cardiomyocytes from EM images. p < 0.001, unpaired t test. (H) Examples of immunostaining of Cav3 in apical and basal myocytes. (I) Morphometric quantification of the regularity of Cav3 staining (from immunostaining micrographs). (J) Quantification of the density of Cav3 staining. N, n = (3) 12/13, NS, unpaired t test. Error bars indicate SEM.

Cell plasma membrane topography images were obtained using SICM, and no gross differences were apparent (Figure 2A). We then quantified the regularity of Z-grooves, periodic invaginations of the plasma membrane, and found no differences (Figure 2B). Basal and apical cells were stained with pyridinium, 4-[2-[6-(dioctylamino)-2-naphthalenyl]ethenyl]-1-(3-sulfopropyl)-, inner salt 157134-53-7 (di-8ANNEPPS) (Figure 2C). T-tubule density and regularity were calculated (Figures 2D and 2E). Basal cells demonstrated more regular T-tubular elements and a greater density of membrane staining in comparison with cells derived from the apical myocardium.

Transmission electron microscopy revealed a greater number of caveolae within basal cardiomyocyte plasma membranes in comparison with apical cells (Figure 2F). Scoring caveola density demonstrated that there were almost 8-fold more caveolae in the basal cardiomyocyte membranes than in the apical ones (Figure 2G). Similarly, in mouse apical myocytes, we found 4-fold fewer caveolae than in basal myocytes (Figures S3A and S3B). Cav3 immunostaining revealed differences in the relative degree of apical and basal organization analogous to those discovered with di-8ANNEPPS (Figures 2H–2J).

### Elimination of Caveolae Equilibrates Apical and Basal Cell Contractile Responses to β_2_AR Stimulation following Conditional Knockout of Cav3 in Mouse Myocardium

Specialized membrane regions such as caveolae and lipid rafts house multiple membrane receptors and signaling effector molecules (Macdougall et al., 2012). Having observed a significant regional difference in the number of caveolae, we sought to assess the role of caveolar compartments in the observed variability in contractile response to β_2_AR stimulation. Methyl-β-cyclodextrin (MCD), which removes cholesterol from the plasma membrane (Macdougall et al., 2012), was able to dramatically reduce the number of caveolae in both basal and apical cardiomyocytes (Figures S4A and S4B).

**Figure 3:**
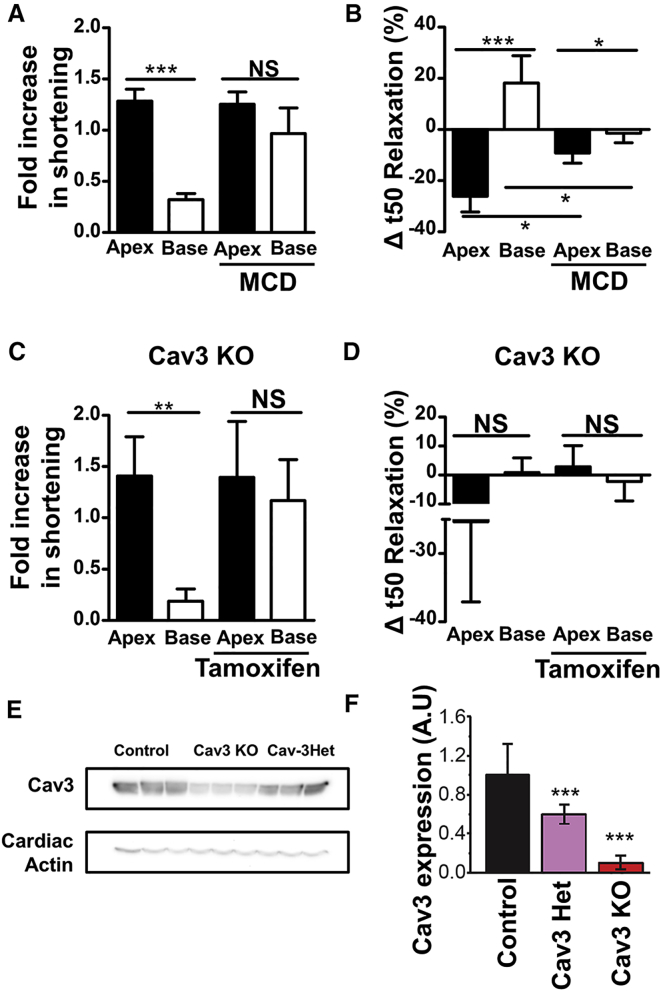
Role of Caveolae in Restraining Contraction of Basal Myocytes following β_2_AR Stimulation (A) Effect of MCD (1–2 mM) pre-treatment on the shortening responses of apical and basal cardiomyocytes from rats following β_2_AR stimulation with 1 μM ISO + 300 nM CGP. N, n = (3) 6/6, p < 0.001, apex versus base, unpaired t test. (B) Effect of MCD pre-treatment on the relative change in relaxation speed of rat apical and basal cardiomyocytes following β_2_AR stimulation. N, n = (3) 6/6, p < 0.05, p < 0.001, unpaired t test. (C) The influence of genetic KO of Cav3 on the shortening responses of apical and basal cardiomyocytes from mice following β_2_AR stimulation. N, n = (3) 6/6, p < 0.01, unpaired t test. (D) Effects of conditional genetic KO of Cav3 on the relative change in relaxation speed of apical and basal cardiomyocytes following β_2_AR stimulation. N, n = (4) 9/10, NS, unpaired t test. (E) Western blot analysis of Cav3 levels in cardiac myocytes isolated following tamoxifen treatment of cav3flox/flox/Mer-αMHC−MerCre+/− mice. (F) Quantification of the amount of Cav3 on the blots in (E) by densitometry. Error bars indicate SEM. See also Figures S2–S4.

We found that, after MCD treatment, the basal cell contractile response to β_2_AR stimulation is equilibrated with that of apical cells (Figure 3A). The baseline contractility of these two cell types appears to be largely unaltered by pre-treatment with MCD (Figure S2B). Interestingly, in apical cells, pre-treatment with MCD reduced the effect of β_2_AR stimulation on relaxation velocity (Figure 3B). In contrast, it accelerates the relaxation of basal cardiomyocytes following β_2_AR stimulation (Figure 3B).

MCD treatment is a coarse method of removing cellular caveolae. To confirm our findings, we utilized apical and basal cells from mice in which Cav3 was knocked down in cardiac myocytes. This removed most cellular caveolae from the Cav3 knockout (KO) basal cardiomyocytes (Figure S3B). After mice had been treated with tamoxifen to remove Cav3 gene expression, both apical and basal cells became equally responsive to β_2_AR stimulation (Figure 3C). Like MCD treatment, following Cav3 knockdown, apical cells did not increase their relaxation velocity in response to β_2_AR stimulation (Figure 3D). Data showing the effectiveness of the tamoxifen-inducible conditional promoter are presented in Figures 3E and 3F. Faithful knockdown of Cav3 mRNA production is demonstrated in both apical and basal cells (Figure S3C). Neither KO of Cav3KO or removal of cholesterol from cell membranes appears to affect T-tubular density or regularity (Figures S4C–S4F).

### β_2_AR-cAMP Responses in Both Plasma Membrane and RII Nanodomains Are Greater in Rat Apical Cardiomyocytes

We measured local cAMP production to assess the relative level of compartmentation in different subcellular nanodomains upon generation of cAMP following β_2_AR stimulation. Apical and basal cardiomyocytes were transfected with either a plasma membrane-targeted (pmEPAC2) or a PKA_RII nanodomain-targeted sensor (RII_EPAC). cAMP production at the plasma membrane following β_2_AR stimulation was found to be greater in apical than in basal cardiomyocytes (Figures S5A and S5B). Basal cells show smaller but substantial cAMP production in the membrane nanodomains. This difference becomes more pronounced when the response is normalized to total cAMP output. A significantly larger difference in cAMP response following β_2_AR stimulation was measured in apical cells by the RII_EPAC sensor than in basal cardiomyocytes (Figure S5C). Normalization of β_2_AR-dependent cAMP response to the total cAMP output clearly shows a higher sensitivity of apical cells in comparison with basal cells in the RII domain (Figure S5D).

### The Removal of Caveolae Increases the Access of β_2_AR-cAMP to Specific Subcellular Nanodomains in Rat Cardiomyocytes

**Figure 4:**
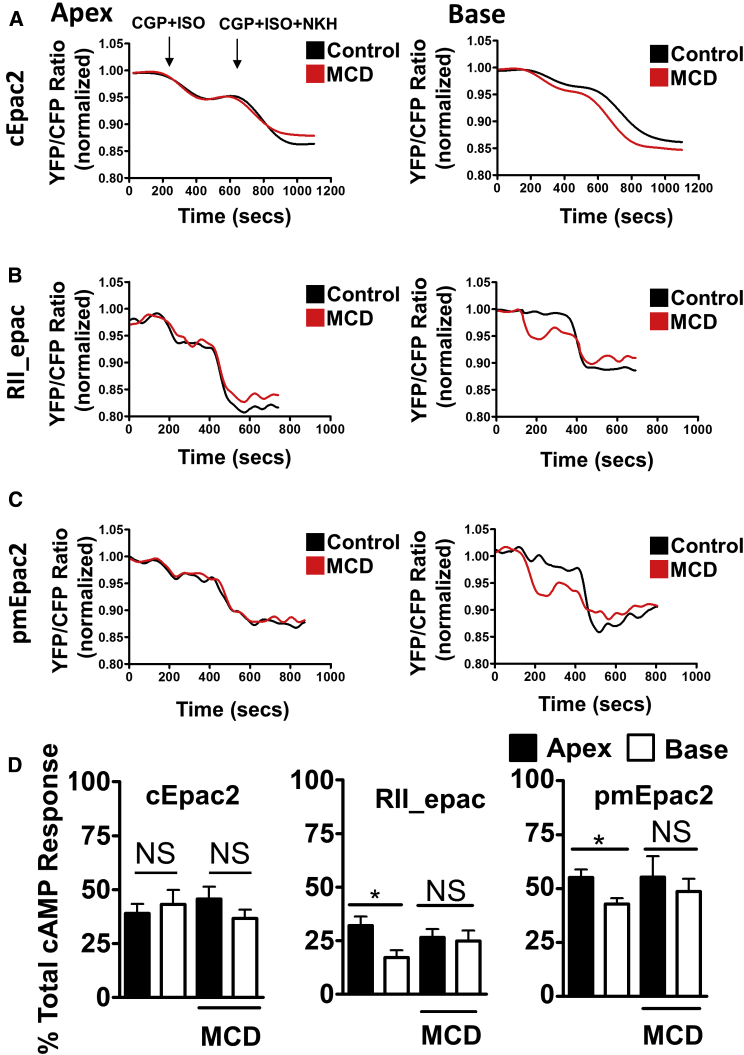
Role of Caveolae/Cholesterol in Compartmentation of β_2_AR Signaling in the Apex and Base (A–C) Representative traces showing rat apical and basal cell cAMP responses with or without MCD (1–2 mM) pre-treatment following β_2_AR stimulation with 100 nM ISO + 100 nM CGP, as measured with a cytosolic (cEPAC2) FRET biosensor (A), in the PKA_RII compartment (RII_EPAC) (B), and in the plasma membrane compartment (pmEPAC2) (C). (D) Quantification of cAMP responses corrected to total cAMP output. Apex/base cEPAC2, N, n = (5) 22/14, (5) 10/7; RII_EPAC, N, n = (5) 20/20, 11/15; and pmEPAC2, N, n = (5) 16/18, 15/15; unpaired t test; ^∗^p < 0.05. Error bars indicate SEM. See also Figures S4 and S5.

We wanted to find out whether the changes in contractile response we observed following the removal of caveolae were due to the altered cAMP access to different nanodomains. Treatment with MCD did not change β_2_AR-dependent cAMP responses measurable in the cytosol of both apical and basal cardiomyocytes (Figures 4A and 4D). Moreover, in apical cells, no significant change is seen in both RII_PKA and membrane compartments (Figures 4B and 4D). In basal cardiomyocytes, a smaller cAMP response was observed following β_2_AR stimulation in either RII or membrane domains (Figures 4C and 4D). However, pre-treating the basal cardiomyocytes with MCD leads to the appearance of a cAMP response in both RII domains (Figure 4B) and at the plasma membrane (Figure 4C). Correction of the β_2_AR-dependent cAMP response to total cAMP also shows that MCD pre-treatment increases the β_2_AR response of basal cardiomyocytes in both RII (Figure 4B) and membrane (Figure 4C) domains, equilibrating the apical and basal responses.

### Inhibition of PDE4 Using Rolipram Enhances β_2_AR-cAMP Responses and Equilibrates Rat Apical and Basal Cell Contractile Responses to β_2_AR Stimulation

**Figure 5:**
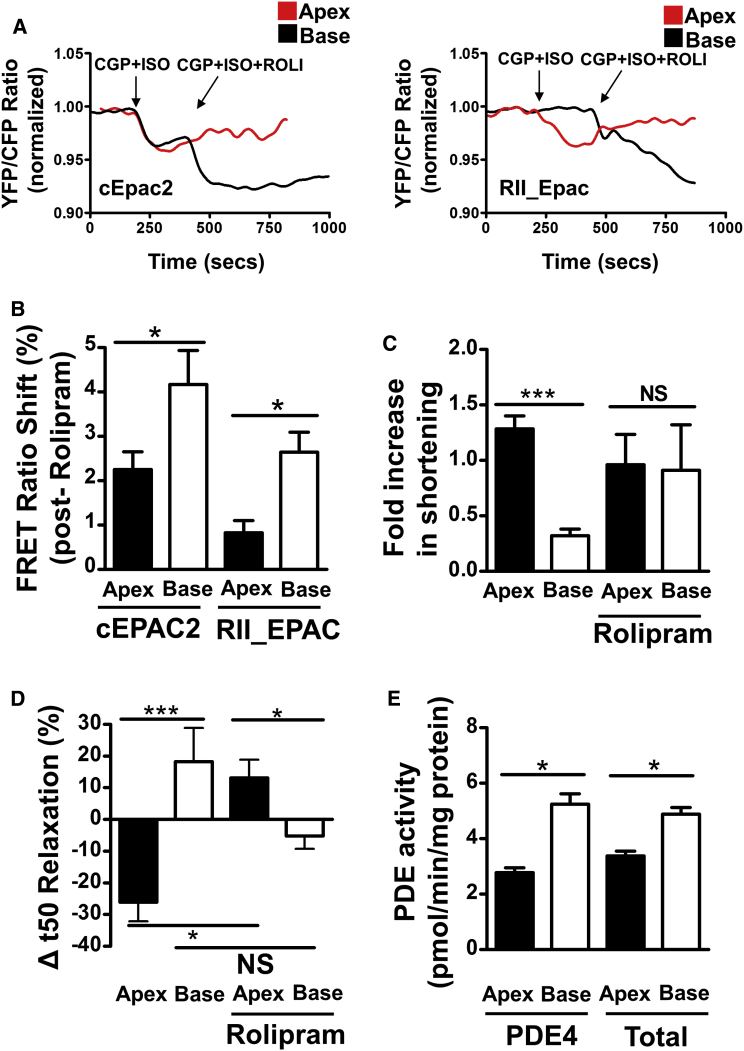
Investigating the Role of PDE4 in Compartmentation of β_2_AR Signaling in the Apex and Base by Inhibition with ROLI (A) Representative trace showing the FRET responses of rat apical and basal cells following the stimulation of β_2_AR (100 nM ISO + 100 nM CGP) and subsequent inhibition of PDE4 by ROLI (5 μM) infusion, measured in cytosolic and RII_PKA compartments. (B) Quantification of the relative increase in FRET response following infusion of ROLI. N, n = (6)21/22, n = 13/13, unpaired t test, p < 0.05. (C) Effect of ROLI pre-treatment on the shortening responses of apical and basal cardiomyocytes following β_2_AR stimulation. N, n = (3)5, unpaired t test, ^∗∗∗^p < 0.001. (D) Effect of ROLI pre-treatment on the relative change in relaxation speed of apical and basal cardiomyocytes following β_2_AR stimulation. Unpaired t test, ^∗^p < 0.05, ^∗∗∗^p < 0.001. (E) Specific PDE4 and total PDE activity in apical and basal cell caveolar membrane preparations. N, n = (6)6, unpaired t test, ^∗^p < 0.05. Error bars indicate SEM. See also Figures S2 and S6.

One way of restricting the production of cAMP in nanodomains is to regulate how fast it is inactivated by PDEs. We used inhibitors of three PDEs known to be expressed in cardiac myocytes to assess the role of each isoform in the control of cAMP within apical and basal cells using a cytosolic FRET sensor. PDE2 and PDE3 activity was not significantly different between the apex and base (Figure S6A). Inhibition of PDE4 with rolipram in apical and basal cells resulted in both cell types displaying increased contractility at baseline (Figure S2C) compared with control values (Figure S2A). However, inhibiting PDE4 alone is not enough to provide maximum contractile output at baseline because, upon β_2_AR stimulation, increased contraction was observed in both apical and basal cells (Figure S2C); this effect is similar to MCD treatment (Figure S2B). PDE4 is considered to predominate in removing cAMP from the plasma membrane and PKA compartments in rodent cardiomyocytes (Richter et al., 2011). We sought to find out whether the observed decrease (as reported in previous figures) in localized cAMP responses to β_2_AR stimulation in basal cells is a result of increased PDE4 activity. We used either the global sensor cEPAC2-camps or the localized sensor RII_EPAC. Perfusion of rolipram in apical cells after β_2_AR stimulation results in little further increase in cAMP in either the cytosol or RII domains; however, basal cells demonstrate an increased β_2_AR-cAMP response under these conditions in both the cytosol and RII domains (Figures 5A and 5B). Normalized values for the β_2_AR-dependent contractile responses (Figure 5C) following PDE4 inhibition are equilibrated in apical and basal cells, which is reminiscent of both MCD treatment (Figure 3A) and Cav3 removal (Figure 3C). Without rolipram, only the apical cells’ relaxation velocity significantly decreased (time to relaxation to 50% of baseline length [R50] increased) after β_2_AR stimulation. In the presence of rolipram, basal cells increased in relaxation velocity after β_2_AR stimulation, but this did not reach statistical significance (Figure 5D).

### PDE4 Localization in Caveolae Contributes to Apical/Basal Differences in Rat Cells

To assess how quickly the cAMP produced following β_2_AR stimulation is depleted in the RII compartment, we looked at the FRET ratio over time following a rapid washout of ISO. The cAMP levels remained elevated longer after stimulation in apical versus basal cells (Figures S6F and S6I). Treatment of both cell types with rolipram (Figures S6G and S6J) or MCD (Figures S6H and S6K) removed this difference and equilibrated the relative persistence of cAMP in these nanodomains. In addition to functional assays in isolated cells, we performed a biochemical assay of the PDE4 activity on isolated caveolin-rich membrane fractions of myocytes. Higher PDE4 activity was observed in basal cells compared with apical cells (Figure 5E). Moreover, the total PDE activity is also higher in the caveola-rich membrane fraction of basal cardiomyocytes compared with apical cells (Figure 5E). A significantly greater amount of PDE4B but not PDE4D (although there is a strong tendency) is apparent in basal buoyant fractions in comparison with those isolated from the apex (Figures S6B and S6E).

### β_2_AR-Dependent Production of cAMP Is Greater in the Apex of a Perfused Mouse Heart Than in the Base

**Figure 6:**
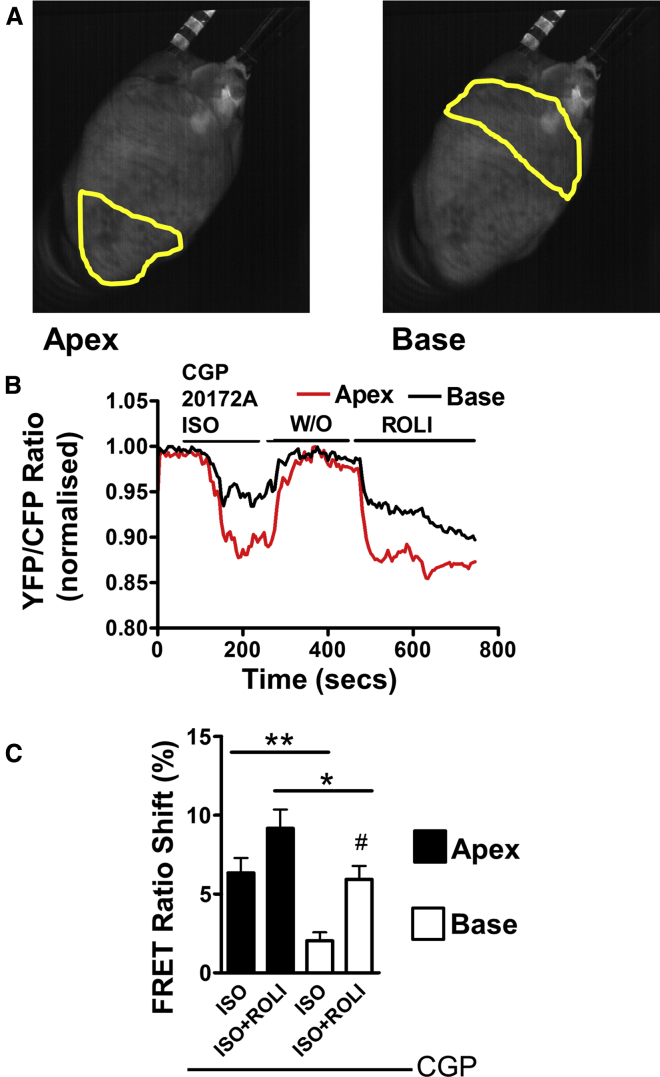
Imaging Regional Differences in β_2_AR-cAMP Responses in a Langendorff-Perfused Mouse Heart (A) Images demonstrating the selection of regions for FRET analysis (these are broadly representative of the regions dissected for cells in other studies) to allow assessment of FRET responses. Images show the yellow fluorescence channel (FRET acceptor). (B) Representative traces presenting the responses of the apical and basal portions of the myocardium under different pharmacologic stimuli. (C) Quantification of FRET responses to β_2_AR stimulation (100 nM ISO + 100 nM CGP) and ROLI (5 μM) from the apical and basal sectors. N, n = (8) 8, p < 0.05, unpaired t test; #p < 0.05, paired t test, CGP+ISO versus CGP+ISO+ROLI steps. Error bars indicate SEM. See also Figure S7.

Having observed the differences of β_2_AR-cAMP signaling compartmentation in apical and basal cardiomyocytes from rat and mouse, we wanted to see whether these differences exist at the level of the whole heart. We monitored cAMP production in Langendorff-perfused hearts isolated from pmEPAC2-camps transgenic mice following baseline measurements and perfusion with CGP and with treatment with ISO to produce a β_2_AR-stimulated cAMP response. After a period of washout, the PDE4 blocker rolipram, along with CGP and ISO, was perfused into the heart to reveal the relative control of cAMP by this PDE subtype following β_2_AR stimulation. Greater FRET responses were measured at the apex in comparison with the base (Figures 6A and 6B) following CGP and ISO stimulation, suggesting a greater β_2_AR response in the apex. Following washout, FRET signals rapidly dissipated in both the apical and basal regions.

Perfusion with CGP+ISO+rolipram then caused a large FRET response in both regions. Following image analysis, a significant difference in β_2_AR-stimulated cAMP responses can be observed between the apex and the base (Figure 6C). A significant difference (paired measurement) was observed between CGP+ISO and CGP+ISO+rolipram (ROLI) steps in the basal segment but not the apical one, suggesting a comparatively greater role for PDE4-mediated cAMP control in this region (Figure 6C). Overall, larger responses were observed at both stages within the apical segment. We repeated this experiment to examine the effect of epinephrine, a more physiologically relevant ligand, delivery into intact perfused pmEpac1 hearts. A significantly greater amount of cAMP is produced in the apical segment in comparison with the basal, after application of this agent (Figure S7).

## Discussion

**Figure 7:**
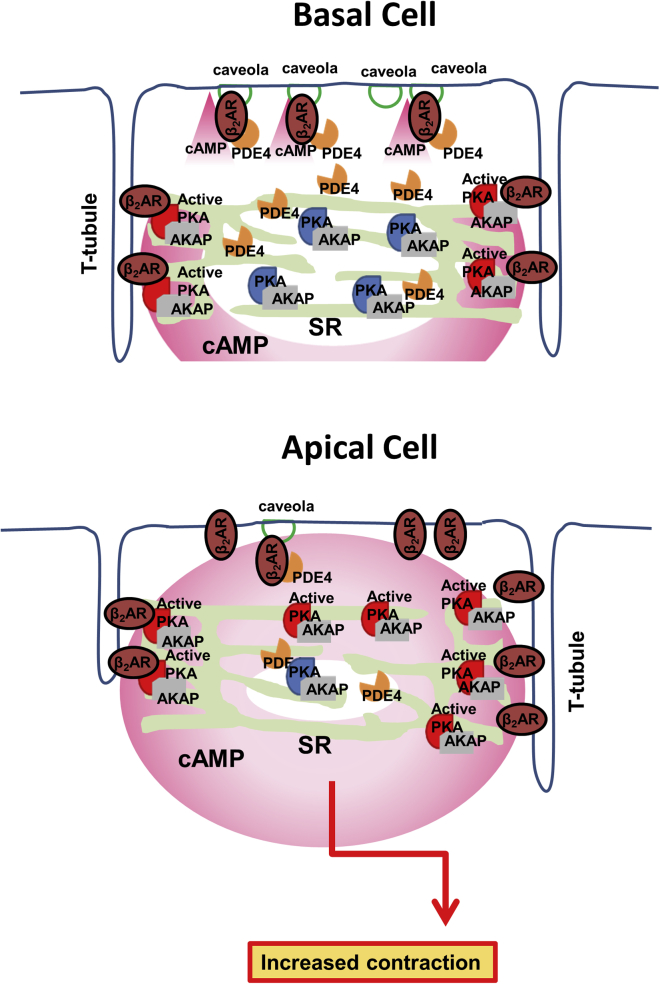
Simplified Depiction of the Differences in β_2_AR Nanodomains in Apical and Basal Cardiac Myocytes Top: the scenario within relatively well organized basal cardiomyocytes. A high degree of membrane organization in basal cardiomyocytes allows tight control of the cAMP level because of enhanced efficacy of PDE4-mediated cAMP hydrolysis. As a result, the cAMP produced by β_2_AR stimulation cannot modulate cell contractility to a great extent. Bottom: the scenario of less organized apical cardiomyocytes. Apical cardiomyocyte membranes lack extensive nanodomain compartmentation, and, as a result, β_2_AR-stimulated cAMP is able to reach deeper from the membrane and increases cellular contractility via PKA-mediated control of the excitation contraction (EC) coupling machinery.

Different regional responses to stress and high catecholamine levels are observed in human hearts after stressful experiences and in animal models of stress or epinephrine infusion. The studies here demonstrate that contractile responses of cardiomyocytes from separate myocardial regions differ following β_2_AR stimulation. The presence of a cAMP response in the cytosol following β_2_AR stimulation does not necessarily result in a contractile response. We present data that suggest that the characteristics of left ventricular cardiomyocyte responsiveness to β_2_AR stimulation are linked to regional origin. The concept central to our hypothesis is that cAMP must be conducted from its region of production (the plasma membrane) to cAMP-dependent effector molecules (the RII domains of PKA). These are spatially separate, so this process may involve transit through a region both separate from production and/or not involved in effector control (the cytosol). Our data demonstrate that less β_2_AR-cAMP is detected in the plasma membrane and PKA-RII nanodomains of basal cardiomyocytes following β_2_AR stimulation compared with apical cardiomyocytes. We propose that regional differences are the result of local heterogeneity in effective cAMP generation and removal. These processes are controlled to a large degree by compartmentation of β_2_AR-cAMP by membrane organization and PDE4. cAMP compartmentation appears to be reliant on the structural organization of the cardiomyocyte, specifically the number of caveolae and the density and regularity of t-tubular structures. As a result of this work, we can propose the schematic illustrated in Figure 7.

In the apical myocardium, agonists of β_2_AR stimulate receptors that are not fully compartmentalized because of the reduction in membrane complexity. As a result, the cAMP is less well controlled by the hydrolytic activity of PDE4. As a result, persistent β_2_AR-cAMP penetrates PKA-RII compartments, and molecular actors within the excitation-contraction coupling system are more effectively phosphorylated. In response, apical cells increase their contractility to a greater degree than basal cardiomyocytes following β_2_AR stimulation. Perhaps even more interestingly, we note that basal cardiomyocytes seem to be actively concentrating PDE4B in their caveolae, which is likely to contribute to the greater compartmentation of cAMP alongside the enhancement of membrane complexity.

Our study shows a clear relationship between the persistence of cAMP within the RII domains and the physiological response of cardiomyocytes to β_2_AR stimulation. Pharmacological interventions that increase cAMP in these domains in comparison with a control also appeared to cause increased inotropic and lusitropic responses, in basal cells in particular. This finding is similar to that found in the work of Macdougall et al. (2012). The selective β_2_AR agonist zinterol elicited cAMP responses in the cardiomyocyte cytosol (measured by the cEPAC2 sensor) but not PKA-RII compartments. These data, from cells isolated as a homogenate from the entire left ventricle, bears similarities with cells isolated from the basal myocardium in our study. On the basis of these data, we hypothesize that the apical myocardium represents a discrete portion of the rat heart from a pharmacological perspective. The question of whether the β_2_AR can control excitation-contraction coupling has been investigated in cardiac cells from different organisms of differing levels of maturity and via different metrics (Balijepalli et al., 2006, Kuschel et al., 1999, Rybin et al., 2003). Studies have assessed the effect of β_2_AR on cell contractility. The findings of these publications have been somewhat divergent, with some reporting a role for β_2_AR in the control of adult cardiomyocyte contractility or members of the excitation-contraction coupling machinery. Equally, earlier studies employed either pertussis toxin (PTX) or 3-isobutyl-1-methylxanthine (IBMX) (Balijepalli et al., 2006, Chen-Izu et al., 2000, Kuschel et al., 1999, Rybin et al., 2003). We suggest that the loss of β_2_AR control/compartmentation brought about by these agents would also make the cells less reflective of the situation *in vivo*. We propose that latent heterogeneity within ventricular cardiomyocytes may have contributed to the differing findings of these studies as well as the occasional employment of either IBMX or PTX.

The necessity for the differences between apical and basal cardiomyocyte populations within the myocardium is unclear. However, computational modeling has revealed that fiber strain and external work are lowest at the apical epicardium and highest at the mid-left ventricular endocardium and basal epicardium (Usyk et al., 2000). For βAR stimulation to optimally increase cardiac output, it can be assumed that the regional functional properties need to be differentially enhanced. Thus, the differences in sympathetic functional responses we report may be necessary to selectively enhance apical function to avoid left ventricular outflow track obstruction. This may especially be true of situations where there are periods of elevated sympathetic activation; for example, exercise or emotional stress.

The apical ventricular wall is thinner than the basal part; this provides a conical structure that efficiently propels blood. The anatomy of the apical myocardium coupled with enhanced adrenergic responsiveness makes it an ideal reservoir of contractile force that can be deployed in stressful situations. Stretching cardiac tissue or cells has been demonstrated to decrease conduction velocity in pathological scenarios and in health (Mills et al., 2011). Membrane caveolae are recruited and removed from the plasma membrane by stretch (Kohl et al., 2003). The presence of extra caveolar membranes and the subsequent tighter control of β_2_AR-dependent cAMP production in the base may be a consequence of this important mechanism. The lower amount of caveolae in the apical cells may reduce membrane capacitance, preventing conduction slowing (Kohl et al., 2003).

There are also notable gradients in innervation within the myocardium. The basal myocardium displays a greater degree of innervation than the apical segment (Kawano et al., 2003). One may hypothesize that the presence of a greater density of nerves may inversely drive the expression and control of β_2_ARs. It has been reported that innervation reduces the number of caveolae in neonatal cardiomyocytes in the proximity of the synapse (Shcherbakova et al., 2007). This is somewhat countervailing the findings of this study. The control of caveolae may be more dynamic than these studies have illustrated and different within the adult myocardium. The differences in the control of β_2_AR may be modified in heart disease, such as Takotsubo syndrome, where regional differences in contractile function following stress or exogenous catecholamine administration are overtly present. Future studies should investigate to what extent the regional heterogeneity described in this study is the result of differences in the physical stresses present in different organ microenvironments. In summary, we present a case of cellular micro-domains building tissue/organ-level differences in physiology, which may be important for the control of physiology of many other organs.

## Experimental Procedures

### Animals

Animal experiments met the criteria of Imperial College London and Animals in Scientific Procedures Act (ASPA) 1986 as well as the 2010/63/EU Directive. Apical and basal cardiomyocytes were isolated from the left ventricles of male Sprague Dawley rats (200–500 g), male/female Cav3KO mice (20–30 g), or male/female β_2_AR KO mice (20–30 g) (Devic et al., 2001) by a Langendorff perfusion method as described previously (Nikolaev et al., 2010, Paur et al., 2012; Figure S1). After brief enzymatic perfusion, the hearts were cut down from the Langendorff system. The atria and right ventricle were excised. The remaining left ventricle was divided into three, and the middle section was discarded. The basal and apical sections were disrupted and shaken separately to yield isolate myocytes from separate regions. The cells from the two populations survived with a rod-shaped morphology beyond 72 hr.

Cav3 conditional KO mice were generated by crossing a mouse line with loxP-flanked exon 2 of Cav3 with an alpha-myosin heavy chain proto-oncogene tyrosine-protein kinase MER Cre recombinase proto-oncogene tyrosine-protein kinase MER (αMHC-MERCreMER) (Sohal et al., 2001) mouse producing a tamoxifen-inducible, cardiac-specific Cav3 KO mouse (Markandeya et al., 2015). Mice were given an intraperitoneal injection of 1 mg tamoxifen/day for 3 consecutive days and sacrificed 21 days post-tamoxifen treatment, and cells were isolated as described above. Hearts from male and female pmEpac2 mice were used for *ex vivo* FRET measurements.

### IonOptix-Based Measurements of Cell Contractility

The fractional shortening of cardiomyocytes was assessed on the day of isolation by the IonOptix method (Westwood, MA, US). Cells were perfused with Krebs-Henseleit (37°C, 95%/5% O_2_:CO_2_) paced at 0.5 Hz (rat) or 1 Hz (mouse). The cells of both species were paced (50 V/pulse width, 2 ms). After a baseline period, β_1_ blockade was achieved by perfusing cells with CGP (300 nM) for 10 min (or β_2_ blockade with ICI). Subsequently, ISO was used to stimulate cells’ β_2_AR (1 μM). Following analysis, the shift in fractional shortening (%FS) and the time to R50 was assessed from ten representative peaks from each phase of pharmacologic treatment.

### Measurement of Intracellular cAMP Responses by FRET Microscopy

Isolated cardiomyocytes were plated on laminin-coated coverslips and cultured for 48–56 hr in M199 medium and transfected with FRET constructs by adenoviral delivery. The cEPAC2-cAMPs and pmEPAC2 adenoviruses were a kind gift from Prof. Martin Lohse (University of Würzburg), and the RII_EPAC sensor was a gift from Prof. Manuela Zaccolo (University of Oxford) (Stangherlin et al., 2011). Cells were analyzed between 44 and 56 hr post-transfection. β_1_AR was blocked by CGP (100 nM), and β_2_AR was then stimulated with ISO (100 nM). Finally, total adenyl cyclase (AC) activity was elicited by NKH477 (5 μM) stimulation to allow correction. Variations of this method were performed. The PDE4 blocker ROLI (10 μM) was substituted for NKH477 following β_2_AR stimulation to reveal PDE4 tone in cardiomyocytes. Erythro-9-(2-hydroxy-3-nonyl)adenine (EHNA) (10 μM) was used to probe the function of PDE2, and milrinone (1 μM) was used to assess the role of PDE3. IBMX-sensitive PDEs were probed by perfusion with IBMX (100 μM). Rapidly washing ISO off following β_2_AR stimulation, with no further stimulation, allowed assessment of the kinetics of cAMP hydrolysis/removal within cells. Images of CFP and yellow fluorescent protein (YFP) fluorescence were acquired using a digital camera and optical microscopy system. The intensities of the images were ratioed, and a bleedthrough factor of 0.956 was subtracted and normalized to baseline. Measurements of FRET shifts were then acquired from curves in a number of ways depending on the property or methodology of interest.

### Measurement of Surface Organization by SICM

SICM measurements were performed as described previously (Nikolaev et al., 2010). The cell surface was scanned to allow visualization of T-tubules via the scanning pipette. Calculation of the Z-groove index was produced by measuring the length of Z-grooves from single SICM images and dividing this by the hypothesized maximal Z-groove length in a 10 × 10 μm scan (50 μm).

### Measurement of FRET Responses in Langendorff-Perfused Hearts

FRET-based measurements were performed in Langendorff-perfused hearts harvested from pmEPAC2-camps transgenic mice as described previously (Wachten et al., 2010). After measurement of baseline activity, hearts were perfused with epinephrine (100 nM) or CGP (100 nM) and then ISO (100 nM) ± ROLI (5 μM) (both from Sigma-Aldrich, St. Louis, MO, USA). We used an imaging system created around a Leica M165FC (Leica Microsystems, Wetzlar, Germany) stereomicroscope (for Langendorff perfusion) to measure FRET responses. The cAMP sensor was excited with a 440-nm light emitting diode (LED) (pE-100, CoolLED, Andover, UK) source. Emission light was split into donor and acceptor channels using a DV2 DualView beamsplitter apparatus equipped with a 565DCXR dichroic mirror and D480/30 and D535/40 emission filters (Photometrics, Tucson, AZ, USA). Images were taken using an optiMOS camera (Photometrics, Tucson, AZ, USA) with MicroManager 1.4 open source imaging software and analyzed by ImageJ (NIH, USA) with in-house plugins. Raw data were corrected offline for the bleedthrough factor of the donor into the acceptor channel (Nikolaev et al., 2010).

### Microscopic Techniques: Confocal and Electron Microscopy

Detailed descriptions of our methodology can be found in the Supplemental Experimental Procedures.

### PDE Activity Assay

A full description of the methodology can be found in the Supplemental Experimental Procedures. We used the method of Head et al. (2005) to allow preparation of caveolar and non-caveolar membrane fractions. Our PDE assay was an adaptation of a previous method (Thompson and Appleman, 1971) to determine the cAMP hydrolysis capacity of tissue preparations.

### Statistical Analysis

Data were tested for normality (Kolmogorov-Smirnov test). Where two unrelated populations were analyzed, statistical difference was determined using an unpaired (two-tailed) Student’s t test. Where more than two unrelated populations were analyzed, one-way ANOVA with a Bonferroni post-test was used. This was performed using GraphPad 4.0 software. In the figure legends, N refers to the number of preparations, and n refers to the number of measurements.

### Data Availability

The data presented in this study are available from the authors upon request.

## Supplemental Information

Document S1. Supplemental Experimental Procedures and Figures S1–S7

Document S2. Article plus Supplemental Information
