# Supplementary material for: Cardiomyocyte Membrane Structure and cAMP Compartmentation Produce Anatomical Variation in β2AR-cAMP Responsiveness in Murine Hearts
Source: Cell Rep. 2018 Apr 10;23(2):459–69. doi: 10.1016/j.celrep.2018.03.053 (PMC5912947; doi:10.1016/j.celrep.2018.03.053)
Supplement: Document S1. Supplemental Experimental Procedures and Figures S1–S7 [file mmc1.pdf]

Cell Reports, Volume *23*

## Supplemental Information

# Cardiomyocyte Membrane Structure and cAMP Compartmentation Produce Anatomical Variation in $\beta_2$AR-cAMP Responsiveness in Murine Hearts

**Peter T. Wright, Navneet K. Bhogal, Ivan Diakonov, Laura M.K. Pannell, Ruwan K. Perera, Nadja I. Bork, Sophie Schobesberger, Carla Lucarelli, Giuseppe Faggian, Anita Alvarez-Laviada, Manuela Zaccolo, Timothy J. Kamp, Ravi C. Balijepalli, Alexander R. Lyon, Sian E. Harding, Viacheslav O. Nikolaev, and Julia Gorelik**

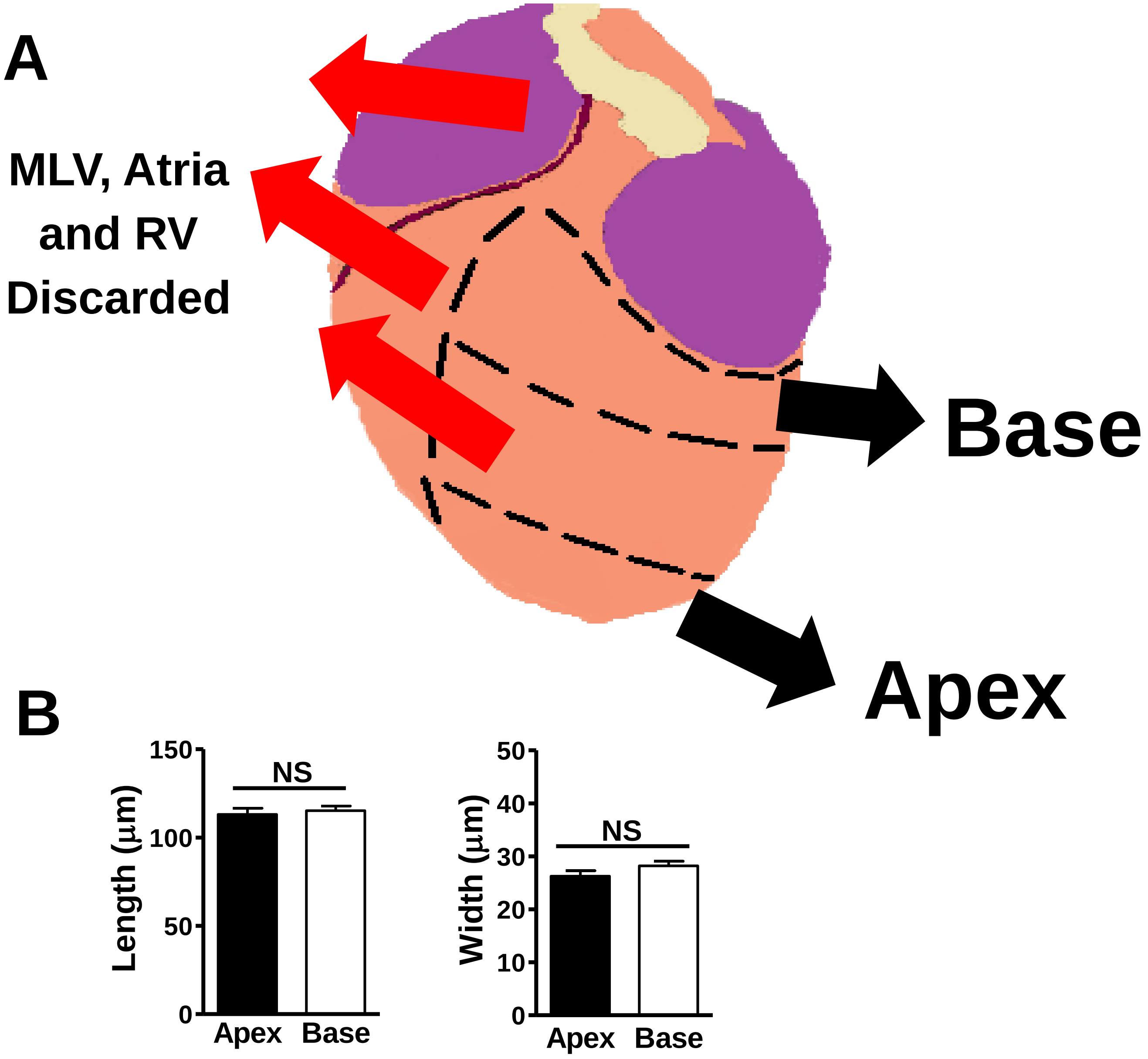


**Figure S1. Diagram demonstrating the isolation of cardiomyocytes from separate myocardial regions. Related to Figure 1.**
a) Following an initial perfusion step with enzymes introduced via Langendorff perfusion, the mid-left ventricle (MLV), atria and right ventricle (RV) are discarded. The basal and apical sections of the left ventricle are then disaggregated in separate vessels to provide pure populations of apical or basal cardiomyocytes. **b)** Cell length and width of apical and basal cardiomyocytes from rat (µm) N,n (3)50.

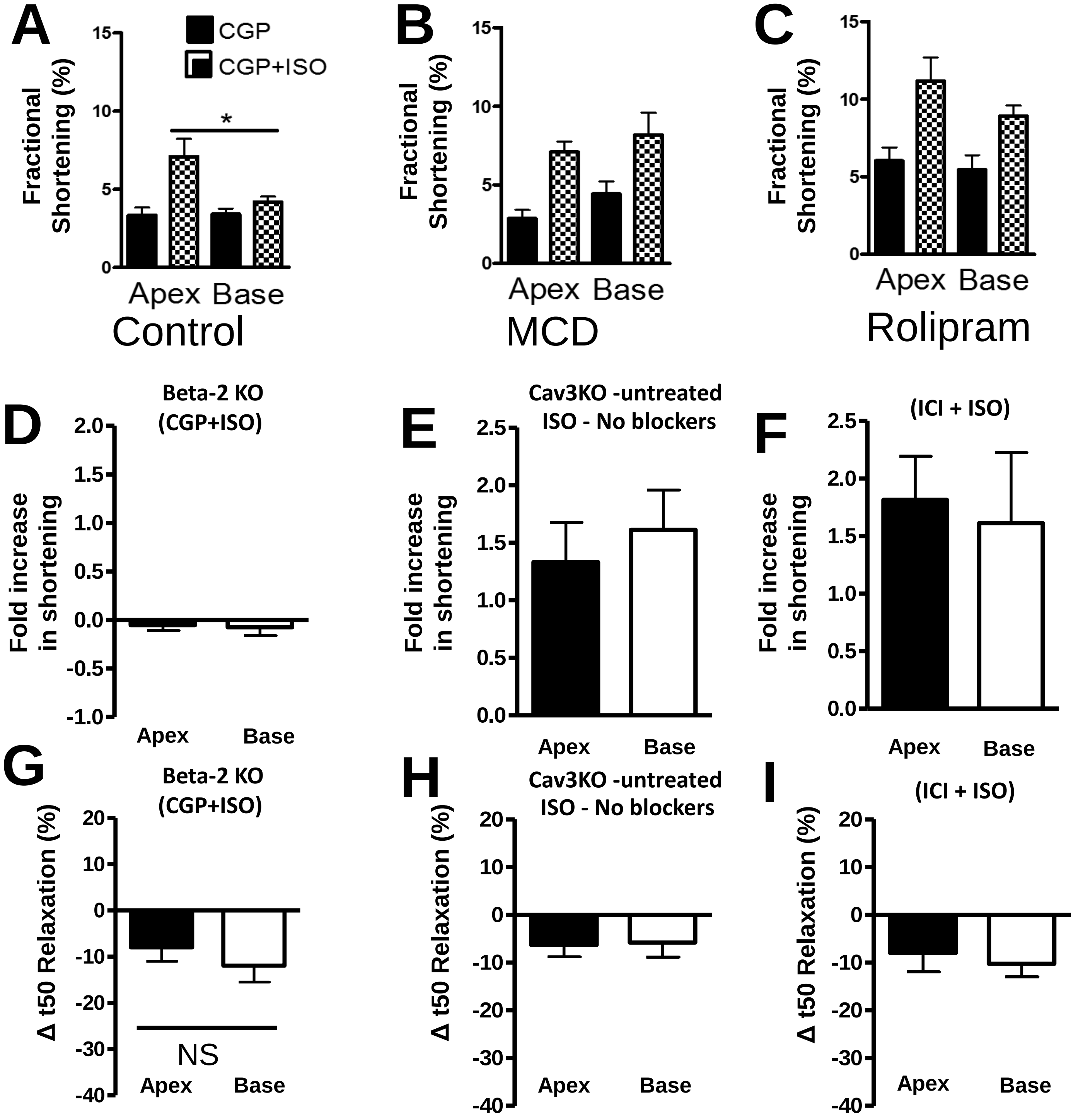


**Figure S2 Contraction of wild type rat myocytes from apex and base, before and after stimulation of $\beta_2$AR. Contraction of $\beta_2$AR KO and Cav3KO (untreated) myocytes. Related to Figure 1, 3 and 5.**
**a.** Untreated cells N,n=(4)11/9; **b.** cells treated with methyl-ß-cyclodextrin N,n=(4)6/9; **c.** or rolipram N,n=(3)6/6. **d**) Fractional shortening response of $\beta_2$AR KO cardiomyocytes CGP+ISO stimulation N, n=(4) 9. **e**) Fractional shortening response of Cav3KO cardiomyocytes following ISO stimulation. N,n=(4) 8-11. **f**) Fractional shortening response of Cav3KO cardiomyocytes following $\beta_1$AR stimulation (ICI118, 551 ($\beta_2$AR blocker) + ISO). N,n=(4) 8 **g**) Change in relaxation response. N,n=(4) 8-11. **h**) Change in relaxation response (ICI118, 551 ($\beta_2$AR) + ISO). N,n=(4) 8 h)). **i**) Change in relaxation response N, n= (4) 9.

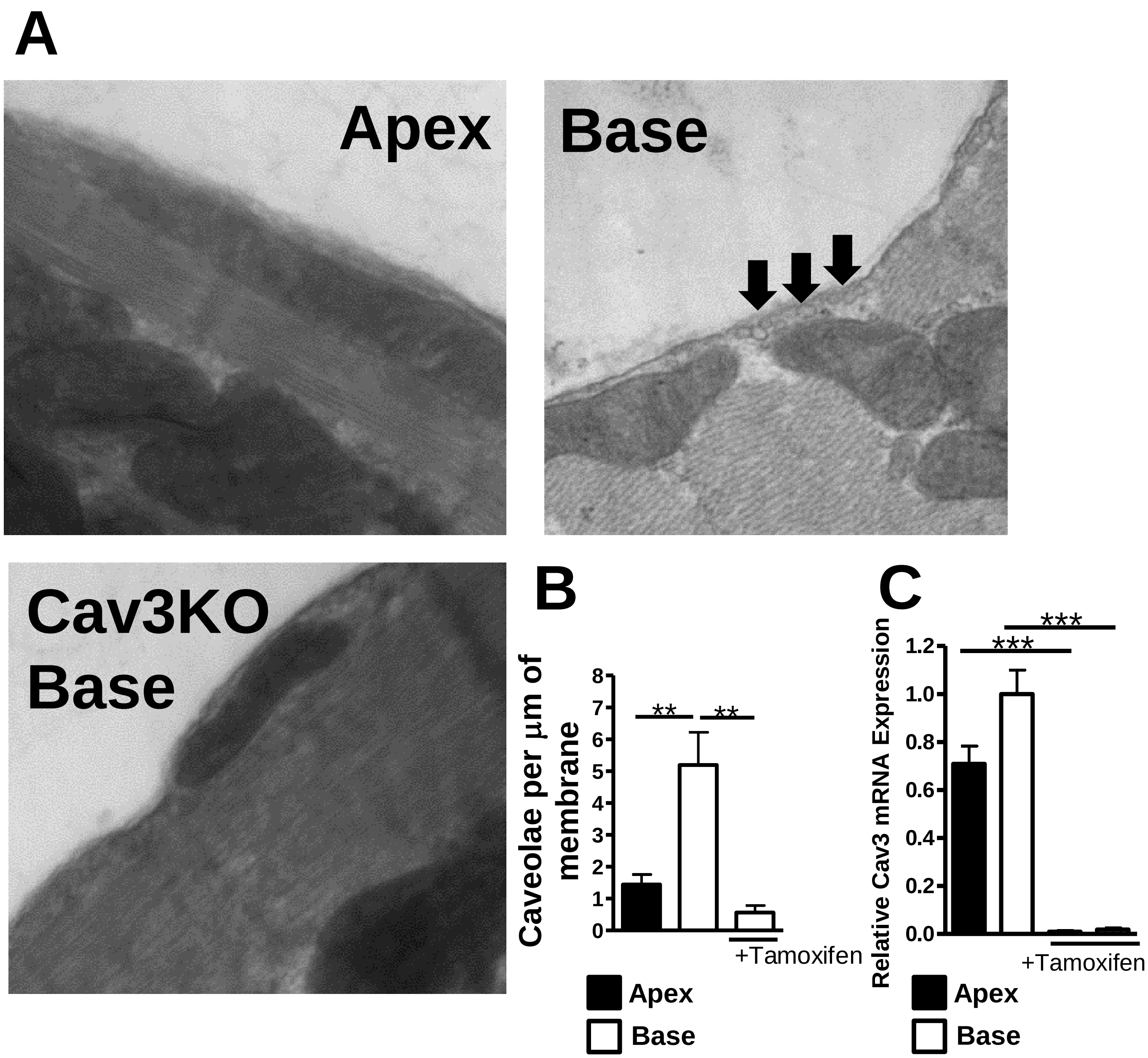


**Figure S3. Characteristics of the Cav-3 KO mouse. Related to Figure 3**.
**a)** Representative electron micrographs of normal apical and basal mouse cardiac myocytes and a basal ventricular myocyte isolated from a Cav3KO mouse. **b**) Quantification of the average amount of caveolae per µm of membrane length (N=10 frames/10 cells; p<0.001) in Cav3KO mice with or without tamoxifen administration **c)** Quantification of the relative expression of Cav3 mRNA in apical and basal myocytes of Cav3KO mice with or without tamoxifen treatment.

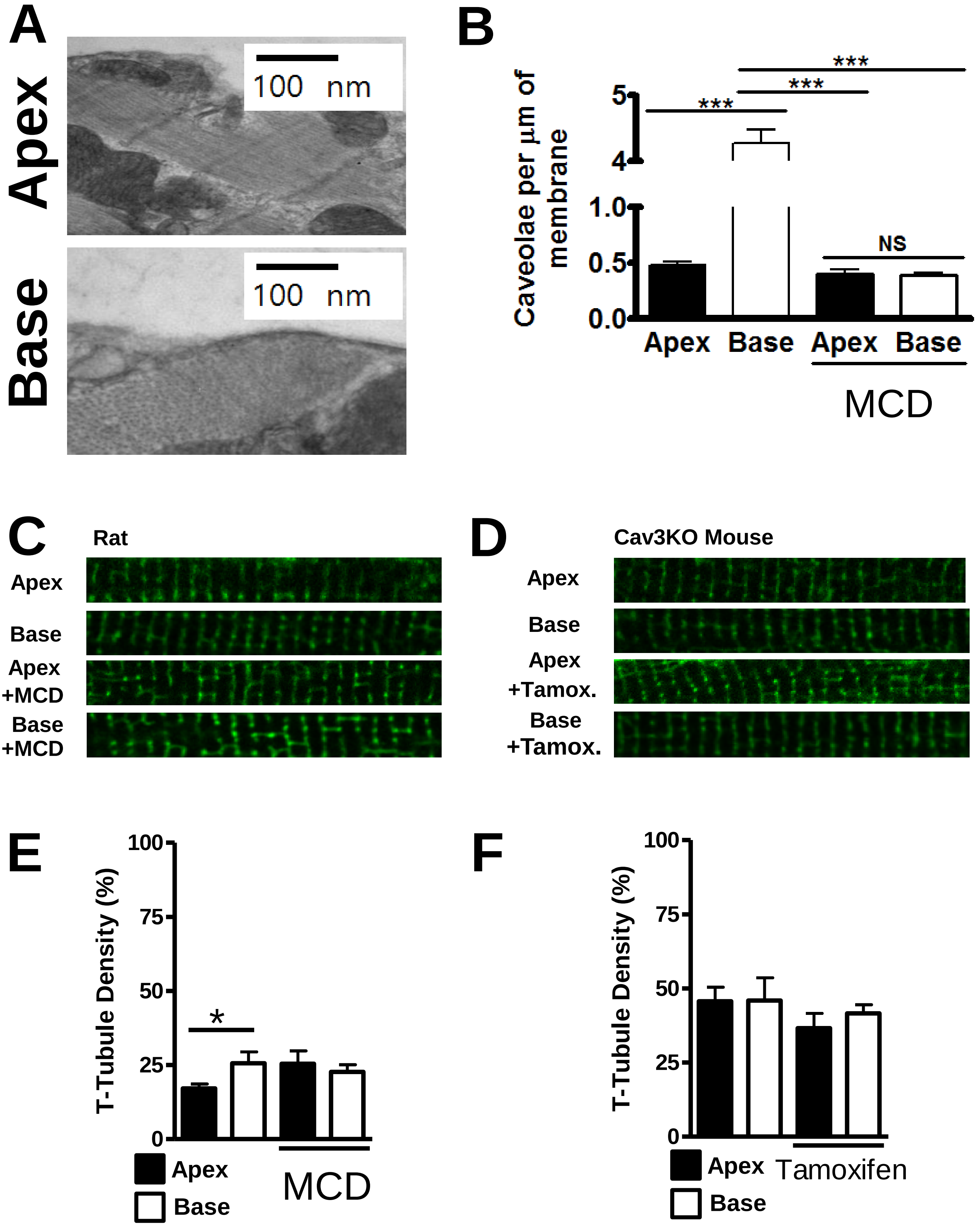


**Figure S4. The removal of caveolae with  MCD and validation of t-tubular density following cell treatment. Related to Figure 3 and 4.**
**a)** Representative electron micrographs of rat apical and basal cardiomyocytes following treatment with methyl-β cyclodextrin **b)**. This leads to the removal of caveolae. **c)** Representative images of apical and basal cells from rat stained with di-8ANNEPPS with or without MCD pre-treatment. **d)** Representative images of apical and basal cells from Cav3KO mice stained with di-8ANNEPPS with or without tamoxifen induced KO of caveolin-3. **e,f)** quantification of t-tubule density.

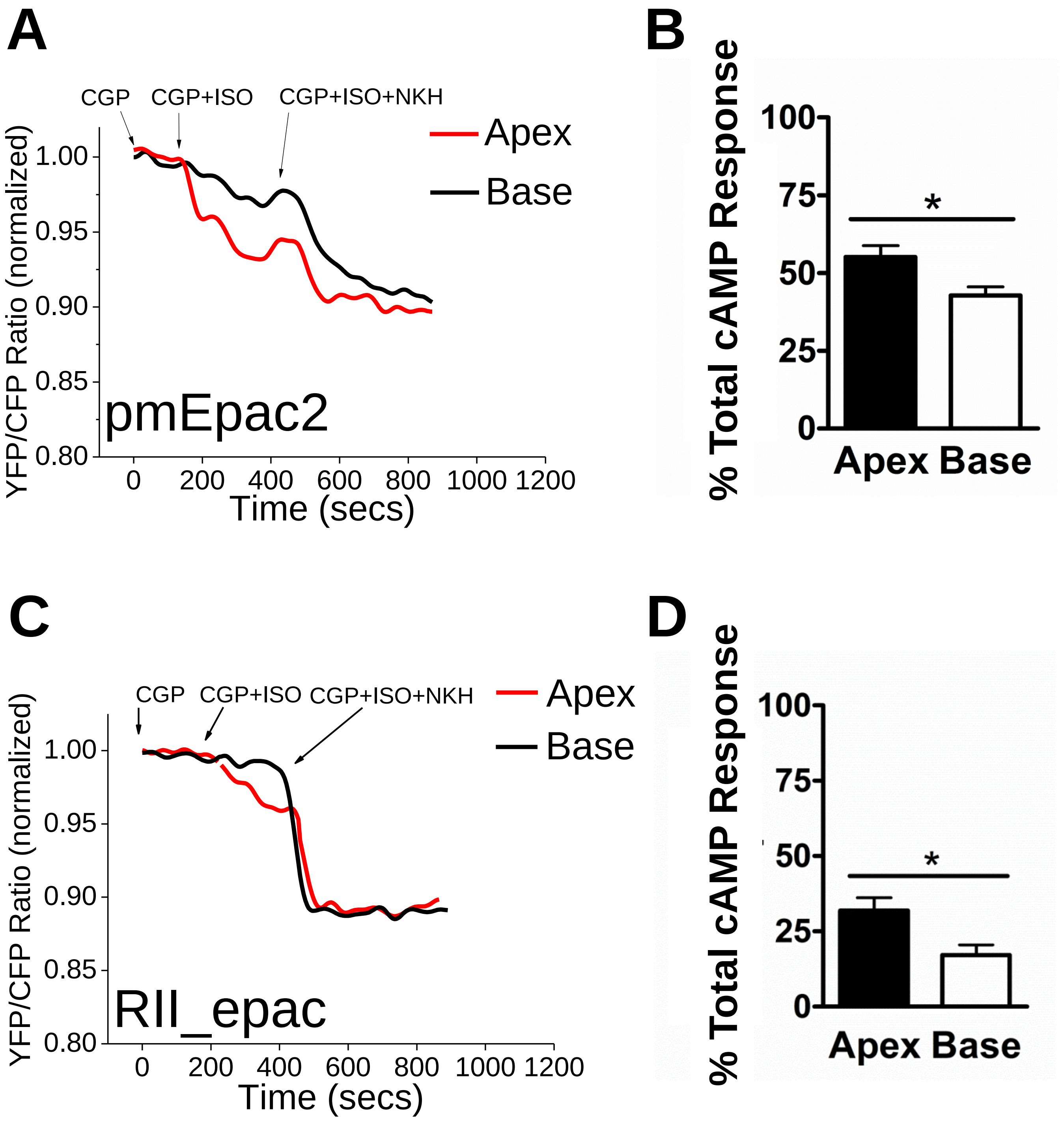


**Figure S5. Stimulated production of cAMP in plasma membrane or RII_Epac nanodomains. Related to Figure 4.** **a)** Representative traces of FRET responses in apical and basal cardiomyocytes from rat as measured by the pmEPAC2 sensor,in the plasma membrane/caveolar nanodomains (**a**, **b**) N,n=(5)21/24, in the PKA_RII nanodomains. (**c**, **d**). N,n=(4)13/13.

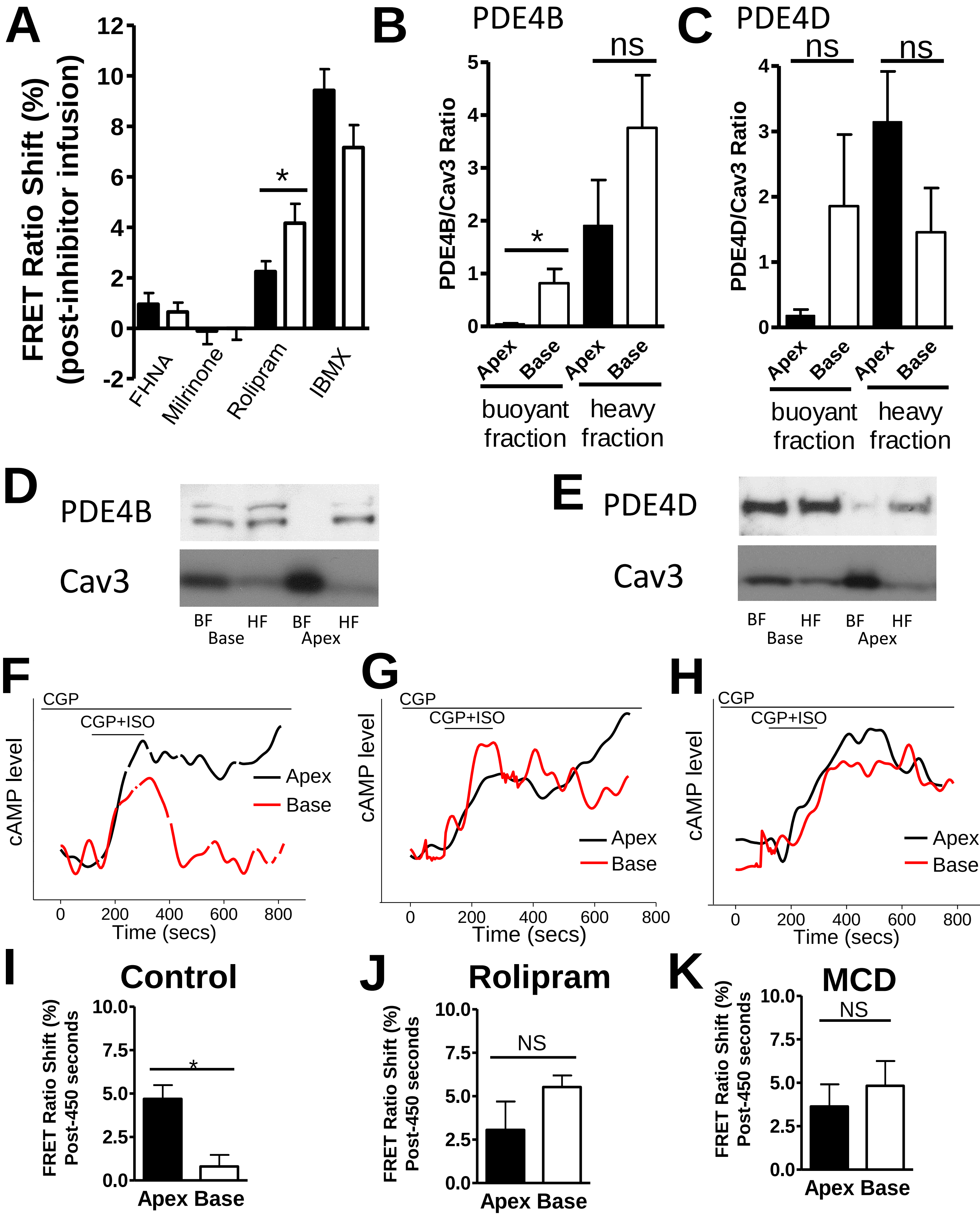


**Figure S6. Increased levels of PDE4 and PDE4 activity are present in apical cardiomyocytes. Related to Figure 5.**
**a**) FRET responses in cEPAC2 transfected cells following $\beta_2$AR stimulation and perfusion with inhibitors of PDE2 (EHNA) N,n=(4)14/14, PDE3 (milrinone 1 μM) N,n=(4)12/14, PDE4 (rolipram 10 μM) N,n=(5)21/24 or general PDE inhibitor (IBMX 100 μM) N,n=(4)12/14. **b, d)** Quantification of PDE4B corrected to caveolin-3 levels (N=3, 4). **c, e)** Quantification of PDE4D corrected to caveolin-3 levels (N=3, 4). The persistence of cAMP in RII compartments of apical and basal myocytes from rat. **f, g, h.** Representative traces of washout experiments with control myocytes from apex and base N,n=(3) 6,4, or cells treated with rolipram N,n=(3) 6,10 and methyl-ß-cyclodextrin N,n=(3) 4,4 ; **i, j, k**. quantification of washout data at 450 seconds from the start of experiment.

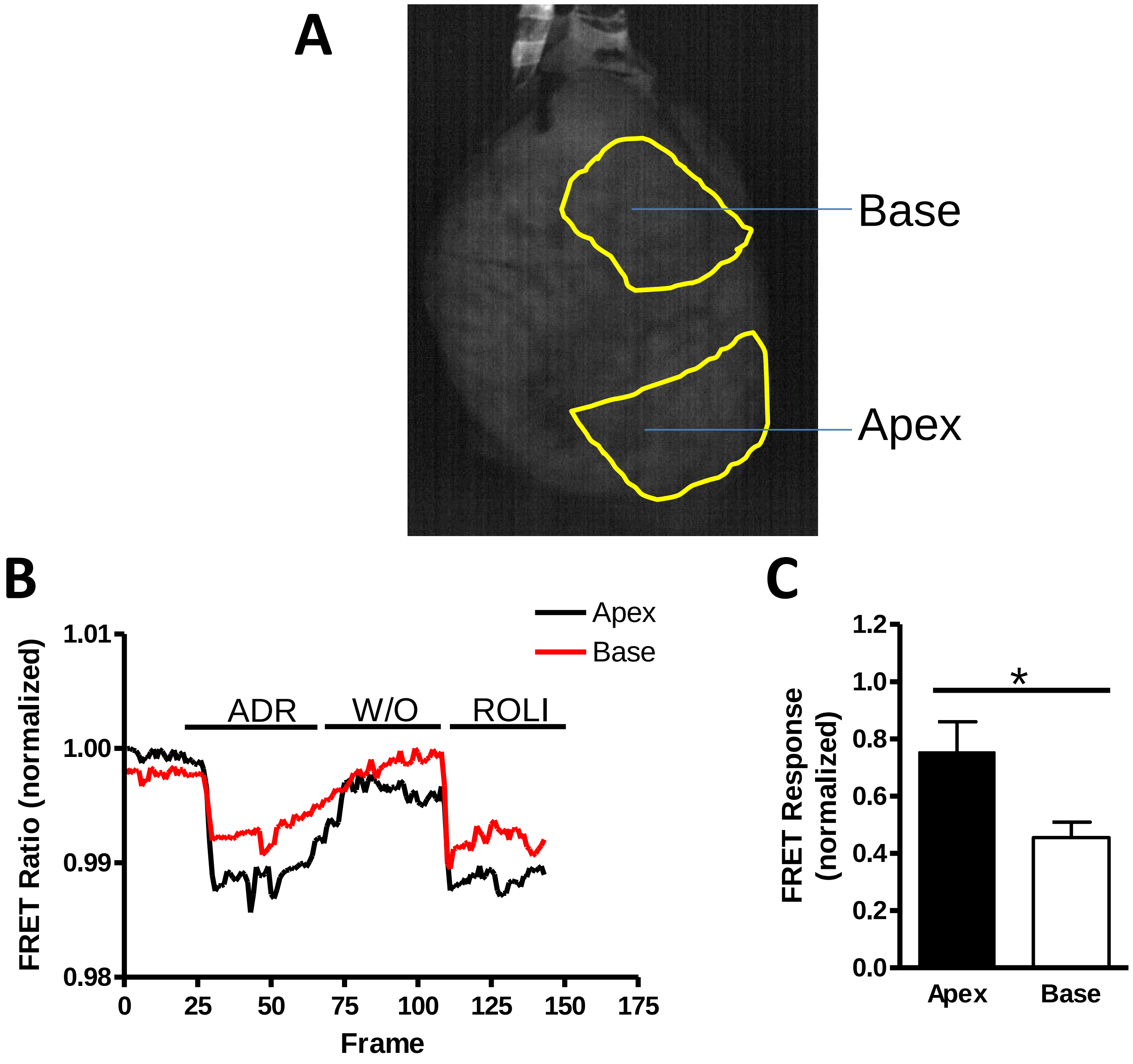


**Figure S7. Regional response of pmEpac1 FRET sensor to epinephrine stimulation. Related to Figure 6.**
**a)** Fluorescent image of the entire heart preparation from the pmEpac1 mouse with apical and basal regions indicated. **b)** Time trace showing the response of the two myocardial regions following infusion of epinephrine (ADR, 100nM), wash out (W/O) and subsequent application of rolipram (ROLI, 10 μM). **c)** Quantification of the average responses of apical and basal regions during epinephrine stimulation phase (N=4).

## Supplementary Methods

### RNA Isolation and Reverse-Transcription Quantitative Polymerase Chain Reaction

Flash frozen tissue from apex or base of WT and Cav3 KO mice were thawed. RNA was isolated using the RNeasy Mini Kit (Qiagen). Complementary DNA (cDNA) was made from 500 ng of isolated RNA using the iScript RT Supermix (Bio-Rad) for reverse transcription. Cav3 mRNA quantification was performed using Taqman Universal PCR Master Mix (Thermo Fisher Scientific) and Taqman Gene Expression Assays for Cav3 (Applied Biosystems, probe Mm01182632_m1) and Gapdh (IDT, probe Mm.PT.39a.1). The expression of Cav3 was normalized to the endogenous control, Gapdh to calculate ΔCq with Cq as the quantification cycle. The relative expression of Cav3 for each group was calculated as 2-ΔCq and normalized to Cav3 expression in the WT base.

### Cell fractionation and Western blotting to establish PDE4 subtype expression

Freshly isolated apical and basal cardiomyocytes were and subsequently fractionated in a sucrose-density column by ultracentrifugation as per the method of Head et al. (See PDE assay section). Buoyant and heavy fractions were retained. These samples were then lysed in 10mM HEPES buffer containing 300mM sucrose, 150mM NaCl, 1mM EGTA, 2mM CaCl2 and 1 % Triton-X and homogenized by pulling the lysate 5x through an insulin cannula (BRAUN, U-40 Insulin, 30Gx1/2, #4001525) followed by 15 min incubation on ice. After centrifugation of the lysate for 5 min at 8000 rpm and 4°C, the supernatant was separated from the pellet and used for protein concentration measurements using a PierceTM BCA Protein Assay kit. Lysates were enriched with 3x reducing buffer (3:1) and 10ug of protein/well were loaded and run on 10% SDS-PAGE gels. For immunoblotting rabbit polyclonal antibodies against PDE4B and 4D were used (1:1000), as well as mouse-monoclonal anti-caveolin-3 (Cav-3) (Applied Biosystems, UK). The optical density of bands were analysed using the freeware ImageJ (available at: https://imagej.nih.gov/ij/ ).

### Determination of membrane regularity and density

Di-8-ANEPPS was used to stain the membranes of isolated cardiomyocytes on day of isolation. These were imaged with an inverted confocal microscope (Zeiss LSM-780). Confocal slices of the cells were generated; T-tubule regularity and density were quantified from 2D images using ImageJ and a bespoke MatLab script (Ibrahim et al., 2010).

### Determination of regularity and density of caveolin-3 containing structures

Cardiomyocytes were fixed with 4% paraformaldehyde on day of isolation. Cell membranes were blocked with 10% FBS and immuno-labelled with mouse-monoclonal anti-caveolin-3 (Cav-3) (Applied Biosystems, UK). Alexa-488 conjugated anti-IgG (donkey anti-mouse) was used to provide secondary labelling. Cells were analysed by confocal microscopy.

### Transmission Electron Microscopy

Apical and basal cardiomyocytes were fixed in glutaraldehyde, washed incorporated into agar and then embedded in araldite. Ultrathin sections 0.1µm thick were stained with uranyl acetate and lead citrate. Images taken with a transmission electron microscope were then used to assess the number of caveolar nanodomains present in cellular membranes. Ten representative fields of view were acquired for each cell and both open and closed caveolae were counted.

**Phosphodiesterase activity assay**

Apical and basal cardiomyocytes were plated on 100mm Laminin coated petri dishes and allowed to attach for an hour and treated as per the method of Head et al. (Head et al., 2005), to allow the preparation of caveolar and non-caveolar membrane fractions. Cells were scraped into a buffer containing Na2CO3 at pH 11.0 The cells were homogenized and the resulting suspension mixed with a sucrose-containing Na2CO3 buffer to create a 45% sucrose buffer. This was transferred to an ultracentrifuge tube and further layers of lower density sucrose buffers (35% and 5%) were applied. These manipulations were performed at 4oC and in the presence of phosphatase and protease inhibitors (Roche, DE). Samples were centrifuged for 16hrs at 4oC and 200,000g. Caveolar and non-caveolar membrane fractions were identified (Head et al., 2005) these preparations were isolated by sequentially aspirating segments of the sucrose gradient. Samples were stored at -80oC until use. PDE assay was an adaptation of a previous method (Appleman and Thompson, 1971) to determine the cAMP hydrolysis capacity of tissue preparations. The caveolar and non-caveolar samples from apical and basal cardiomyocytes were used for a PDE activity assay. Samples were exposed to Tritiated 3’-5’ cAMP with or without 10µM rolipram (PDE4 specific inhibitor) or 100 µM IBMX (non-specific inhibitor) for 60 minutes. The assay was terminated via boiling the mixture for 1 minute. After a brief cool-down period, the mixture was exposed to snake venom nucleotidase for 25 minutes at 30oC. The reaction mix was then loaded on self-made flow-through columns containing 100mg of Bio-Rad resin, AG1-X2, 200-400 mesh. The amount of 3H-Adenosine left in the eluent was then quantified by liquid scintillation as a measure of the cAMP hydrolysis capacity of the preparation and the relative ability of PDE blockade to interfere with this.
