# Supplementary material for: Cardiomyocyte Membrane Structure and cAMP Compartmentation Produce Anatomical Variation in β2AR-cAMP Responsiveness in Murine Hearts
Source: Cell Rep. 2018 Apr 10;23(2):459–69. doi: 10.1016/j.celrep.2018.03.053 (PMC5912947; doi:10.1016/j.celrep.2018.03.053)
Supplement: Document S2. Article plus Supplemental Information [file mmc2.pdf]

Cell Reports

Article

Cardiomyocyte Membrane Structure and cAMP Compartmentation Produce Anatomical Variation in β_2AR-cAMP Responsiveness in Murine Hearts

Graphical Abstract

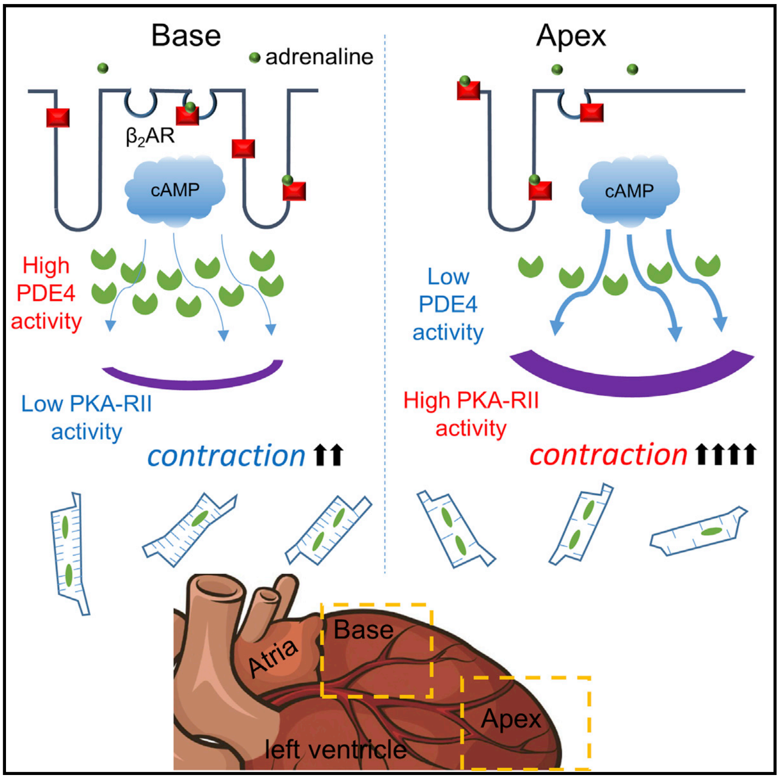

Highlights

- Cardiomyocyte membrane organization varies in degree between regions of the heart
- Differences in structural organization affect adrenergic signaling via β_2AR
- Reduced organization allows β_2AR-cAMP to influence contractility in myocardial apex
- Variability in cell structure may allow differential response of heart regions

Authors

Peter T. Wright, Navneet K. Bhogal, Ivan Diakonov, ..., Sian E. Harding, Viacheslav O. Nikolaev, Julia Gorelik

Correspondence

j.gorelik@imperial.ac.uk

In Brief

Wright et al. present evidence that cardiomyocyte membrane organization (T-tubule regularity and caveolar number) varies between myocardial regions. The reduced membrane organization of cells from the myocardial apex allows β_2AR-cAMP to influence PKA_RII domains. As a result, β_2AR stimulation enhances apically but not basally derived cardiomyocyte contractility.

Wright et al., 2018, Cell Reports *23*, 459–469
April 10, 2018 © 2018 The Authors.
https://doi.org/10.1016/j.celrep.2018.03.053

CellPress

OPEN ACCESS
CellPress

Cardiomyocyte Membrane Structure and cAMP Compartmentation Produce Anatomical Variation in β_2AR-cAMP Responsiveness in Murine Hearts

Peter T. Wright,[1] Navneet K. Bhogal,[1] Ivan Diakonov,[1] Laura M.K. Pannell,[1] Ruwan K. Perera,[2] Nadja I. Bork,[2] Sophie Schobesberger,[1,2] Carla Lucarelli,[1,3] Giuseppe Faggian,[3] Anita Alvarez-Laviada,[1] Manuela Zaccolo,[4] Timothy J. Kamp,[5] Ravi C. Balijepalli,[5] Alexander R. Lyon,[1,6] Sian E. Harding,[1] Viacheslav O. Nikolaev,[2] and Julia Gorelik[1,7,*]

[1]Myocardial Function, National Heart and Lung Institute, Imperial College London, ICTEM, Hammersmith Hospital, Du Cane Road, London W12 0NN, UK
[2]Institute of Experimental Cardiovascular Research, University Medical Center Hamburg-Eppendorf, 20246 Hamburg, Germany
[3]Department of Cardiac Surgery, University of Verona School of Medicine, Azienda Ospedalieria Universitaria Integrata, Borgo Trento Piazzale A. Stefani, 37126 Verona, Italy
[4]Department of Physiology, Anatomy and Genetics, University of Oxford, Oxford OX1 3QX, UK
[5]Department of Medicine, University of Wisconsin Madison, 1111 Highland Ave., Madison, WI 53705-2275, USA
[6]NIHR Cardiovascular Biomedical Research Unit, Royal Brompton Hospital, London SW7 3AZ, UK
[7]Lead Contact
*Correspondence: j.gorelik@imperial.ac.uk
https://doi.org/10.1016/j.celrep.2018.03.053

SUMMARY

Cardiomyocytes from the apex but not the base of the heart increase their contractility in response to β_2-adrenoceptor (β_2AR) stimulation, which may underlie the development of Takotsubo cardiomyopathy. However, both cell types produce comparable cytosolic amounts of the second messenger cAMP. We investigated this discrepancy using nanoscale imaging techniques and found that, structurally, basal cardiomyocytes have more organized membranes (higher T-tubular and caveolar densities). Local membrane microdomain responses measured in isolated basal cardiomyocytes or in whole hearts revealed significantly smaller and more short-lived β_2AR/cAMP signals. Inhibition of PDE4, caveolar disruption by removing cholesterol or genetic deletion of Cav3 eliminated differences in local cAMP production and equilibrated the contractile response to β_2AR. We conclude that basal cells possess tighter control of cAMP because of a higher degree of signaling microdomain organization. This provides varying levels of nanostructural control for cAMP-mediated functional effects that orchestrate macroscopic, regional physiological differences within the heart.

INTRODUCTION

The mammalian heart is a complex 3D structure. The ventricular myocardium is composed of myofibrils, which form layers (Lunkenheimer et al., 2006). Previous research has generally investigated electrophysiological differences between the epicardial and endocardial layers of the ventricles (Stankovicova et al., 2000; Szentadrassy et al., 2005). There is a limited understanding of regional differences, longitudinally, between the apical and basal myocardium. Historically, research into G-protein-coupled receptor (GPCR) function in isolated cardiomyocytes has not accounted for the potential heterogeneity of cardiomyocytes in healthy tissue. Cardiac beta-adrenoceptor (βAR) density is relatively increased in the basal myocardium of dogs (Kawano et al., 2003). A differential "apico-basal" gradient of sympathetic innervation is observed in human samples (Mori et al., 1993). The β-adrenergic responsiveness of the apical myocardium is higher than the basal in rat (Heather et al., 2009), feline (Lathers et al., 1986), and rabbit hearts (Mantravadi et al., 2007). Enhancement of sympathetic input into specific cardiac regions, although possibly supporting efficient cardiac function, may make the apex vulnerable to toxic catecholamine levels during severe stress.

Infusion of the non-specific βAR agonist isoproterenol (ISO) is a classical method of inducing infarct-like damage and chronic heart failure in small animal models. This selectively damages the apex (Rona et al., 1959). In recent years, Takotsubo syndrome (TTS), has been described (Lyon et al., 2016). Multiple preclinical models of TTS, which predominantly afflicts the apex, have been published, involving similar methodologies (Paur et al., 2012; Shao et al., 2013a, 2013b). Previously, we discovered that isolated cardiomyocytes from the apex of rat hearts increased their fractional shortening to a much greater degree than those isolated from the basal segment following β_2AR stimulation (Paur et al., 2012). However, real-time measurements of cytoplasmic β_2AR-cyclic AMP (cAMP) responses were not different between apical and basal cells. This suggested that differences in regional contractile response were not simply the result of an increased density of β_2AR in the apical cardiomyocytes. In this paper, we investigate the role of cAMP control and compartmentation in this phenomenon.

To control contractility in cardiomyocytes, cAMP must engage a protein kinase A (PKA)-dependent (Bers, 2002) signaling

This is an open access article under the CC BY license (http://creativecommons.org/licenses/by/4.0/).

OPEN
ACCESS
CellPress

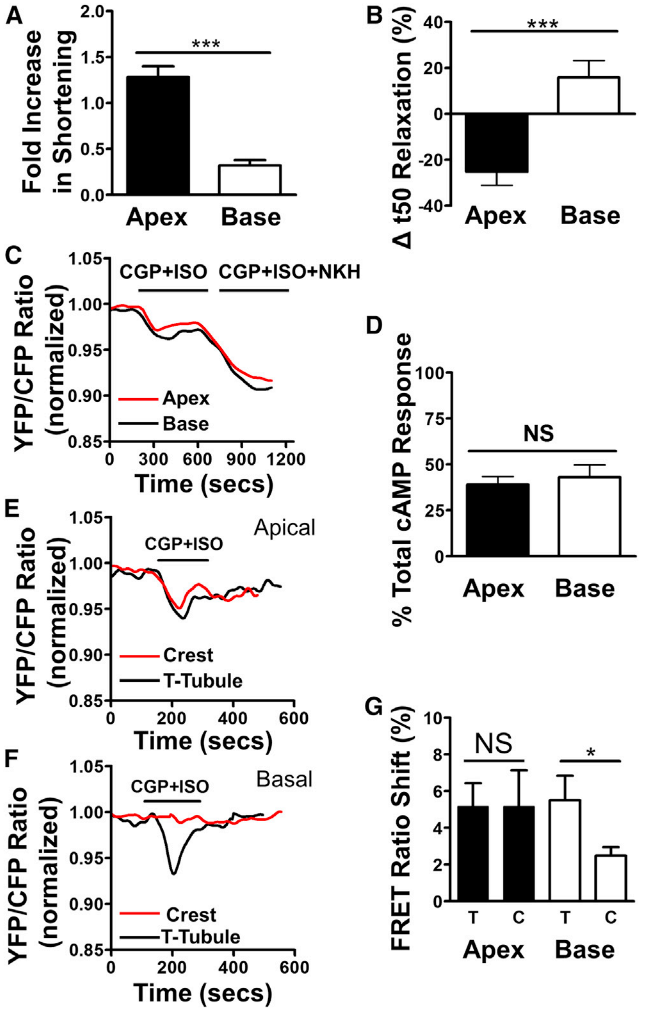

Figure 1. Contraction and cAMP Production in Response to β_2AR Stimulation (Rat Apical and Basal Cells)

(A) Cell shortening increases after β_2AR stimulation (1 μM ISO + 300 nM CGP). N, n = (6) 11/9, $p < 0.001$, apex versus base, unpaired t test.

(B) Relative change in speed of relaxation. N, n = (6) 11/9, $p < 0.001$, apex versus base, unpaired t test.

(C) Representative traces showing the β_2AR FRET response measured by the cytosolic cAMP sensor cEPAC2-camps in apical and basal cardiomyocytes.

(D) Calculation of the amount of cAMP production after β_2AR stimulation relative to absolute maximum of production after stimulation with NKH477. N, n = (10) 36/34, not statistically significant (NS), apex versus base, unpaired t test.

(E and F) Example traces of FRET responses after local stimulation of T-tubules or crest regions in apical (E) or basal (F) cells.

(G) Comparison of apical (tubule/crest, N, n = (9) 15/13, NS) and basal (tubule/crest, N, n = (10) 21/20, $p < 0.05$, unpaired t test) responses.

Error bars indicate SEM. See also Figure S2.

pathway. Because multiple GPCR-based pathways acting via G_s exist in cardiomyocytes, different pools of PKA bound to different A kinase anchoring proteins (AKAPs) are present (Di Benedetto et al., 2008). This allows selective, specific activation of different cell effector systems by different pools of PKA (Carr et al., 1992). The protein kinase A regulatory subunit 2 (PKA_RII) domain controls cardiomyocyte contractility. In this paper, we make use of a fluorescence resonance energy transfer (FRET)-based cAMP sensor that includes a segment of the RII regulatory peptide, allowing it to be localized with membrane-bound PKA and, thus, measure cellular cAMP responses in this cellular region. We also probe the membrane-based control of cAMP by using a plasma membrane-localized FRET sensor (pmEpac2). The cardiomyocyte membrane provides the structural basis for information processing via GPCRs (Di Benedetto et al., 2008; Buxton and Brunton, 1983). The coherence and specificity of βAR signaling is produced by the rapid inactivation or degradation of secondary messengers (Yang et al., 2014; Nikolaev et al., 2006a) as well as removal of the receptor (Liu et al., 2012) and dephosphorylation of PKA targets (Macdougall et al., 2012). Elements of the cardiomyocyte plasma membrane are involved in receptor recycling and provide a mechanism by which phosphodiesterases (PDEs) are made more efficient (Willoughby et al., 2006). The transverse (t)-tubular system and caveolae are the most well characterized organizational elements within the cardiomyocyte membrane with respect to signal transduction (Nikolaev et al., 2010; Wright et al., 2014; Razani et al., 2002; Head et al., 2005). β_2ARs have been shown to be localized within caveolae in cardiac tissue and cells. Removal of β_2ARs from caveolae via removal of cholesterol or caveolin-3 (Cav3) results in enhancement of β_2AR-mediated contractile responses (Macdougall et al., 2012).

In this paper, we demonstrate that apical cardiomyocytes from rat and mouse hearts exhibit greater contractile responses following β_2AR stimulation than those from the base. We show regional differences in membrane organization and a subsequent increase in the stringency of the control of local cAMP microdomains in basal cardiomyocytes by Cav3-associated PDE subtype 4 activity. This has the potential to explain the increased sensitivity of the apical myocardium to circulating catecholamines in pathology and is an example of nanostructural changes in cells leading to differential regional effects on function within organs.

RESULTS

Rat Apical Cardiomyocytes Display Increased Responsiveness to β_2AR Stimulation Compared with Basal Cardiomyocytes from the Same Heart

We isolated cardiomyocytes from the apex and base of the left ventricle of the rat heart (Figure S1A); their gross morphology does not differ (Figure S1B). Contraction was measured under stimulation (0.5 Hz, 50 V). Apical cardiomyocytes display a greater increase in their shortening after β_2AR stimulation than cells derived from the basal myocardium. Normalizing this increase to baseline as well as uncorrected fractional shortening data (Figure 1A and Figure S2A) reveal a significant difference between the responses of apical and basal cardiomyocytes. Apical cardiomyocytes also relax with higher velocity (Figure 1B), taking less time to reach 50% diastolic length after β_2AR stimulation. We confirmed that CGP20712A (CGP)+ISO specifically stimulate β_2AR using β_2AR knockout mice (Figures S2D and S2G). We also stimulated apical and basal cells with ISO alone (Figures S2E and S2H) and with ICI118,551 (ICI)+ISO to

specifically stimulate β_1AR (Figures S2F and S2I). These treatments stimulated the contraction of both cell types to the same degree (Figures S2D–S2I).

β_2AR-Dependent cAMP Levels Are Equal in the Cytosol of Apical and Basal Cardiomyocytes from Rats

We studied β_2AR-dependent cAMP production in cells isolated from the apical or basal myocardium of rats, which were transfected with cytosolic FRET sensor Epac2-based cytosolic cAMP sensor (cEPAC2-camps) (Nikolaev et al., 2006b). Similar levels of cAMP were found to be induced in the cytoplasm of apical and basal cells following β_2AR stimulation (Figures 1C and 1D). The data in Figure 1D are expressed as a percentage of total cAMP production capacity following stimulation of adenylate cyclase activity (via G_s protein stimulation with the forskolin analog NKH477). No statistically significant differences were observed between β_2AR-dependent cAMP levels in apical and basal myocytes.

Stimulated β_2AR-cAMP Responses Are Observed to Be Equal on the T-Tubule and Sarcolemmal Crests of Apical but Not Basal Rat Cardiomyocytes

We have previously demonstrated that, in rat ventricular myocytes, β_2AR-dependent cAMP production is mostly localized to T-tubules, in contrast to β_1AR-dependent cAMP production (Nikolaev et al., 2010). We sought to determine whether there is a difference in the micro-domain response of local β_2AR stimulation within apical and basal myocytes. Initially, scans of cellular topography were acquired using scanning ion conductance microscopy (SICM). β_2ARs were then stimulated in either T-tubule openings or sarcolemma crests via the local application of ISO through the SICM nanopipette in the presence of selective blockade of β_1ARs by CGP in the bath solution. We then measured the relative production of cAMP with the cytosolic FRET sensor cEPAC2-camps in the cell cytosol. We found that, in apical myocytes, β_2AR-dependent cAMP is produced at a similar level in T-tubular and crest regions. However, in basal cells, a higher level of β_2AR-dependent cAMP was produced in T-tubules relative to cell crests (Figures 1E–1G).

Basal Cardiomyocytes from Rats Exhibit a Greater Degree of Membrane Organization in Comparison with Apical Cells

Cell plasma membrane topography images were obtained using SICM, and no gross differences were apparent (Figure 2A). We then quantified the regularity of Z-grooves, periodic invaginations of the plasma membrane, and found no differences (Figure 2B). Basal and apical cells were stained with pyridinium, 4-[2-[6-(dioctylamino)-2-naphthalenyl]ethenyl]-1-(3-sulfopropyl)-, inner salt 157134-53-7 (di-8ANNEPPS) (Figure 2C). T-tubule density and regularity were calculated (Figures 2D and 2E). Basal cells demonstrated more regular T-tubular elements and a greater density of membrane staining in comparison with cells derived from the apical myocardium.

Transmission electron microscopy revealed a greater number of caveolae within basal cardiomyocyte plasma membranes in comparison with apical cells (Figure 2F). Scoring caveola density demonstrated that there were almost 8-fold more caveolae in the basal cardiomyocyte membranes than in the apical ones (Figure 2G). Similarly, in mouse apical myocytes, we found 4-fold fewer caveolae than in basal myocytes (Figures S3A and S3B). Cav3 immunostaining revealed differences in the relative degree of apical and basal organization analogous to those discovered with di-8ANNEPPS (Figures 2H–2J).

Elimination of Caveolae Equilibrates Apical and Basal Cell Contractile Responses to β_2AR Stimulation following Conditional Knockout of Cav3 in Mouse Myocardium

Specialized membrane regions such as caveolae and lipid rafts house multiple membrane receptors and signaling effector molecules (Macdougall et al., 2012). Having observed a significant regional difference in the number of caveolae, we sought to assess the role of caveolar compartments in the observed variability in contractile response to β_2AR stimulation. Methyl-β-cyclodextrin (MCD), which removes cholesterol from the plasma membrane (Macdougall et al., 2012), was able to dramatically reduce the number of caveolae in both basal and apical cardiomyocytes (Figures S4A and S4B).

We found that, after MCD treatment, the basal cell contractile response to β_2AR stimulation is equilibrated with that of apical cells (Figure 3A). The baseline contractility of these two cell types appears to be largely unaltered by pre-treatment with MCD (Figure S2B). Interestingly, in apical cells, pre-treatment with MCD reduced the effect of β_2AR stimulation on relaxation velocity (Figure 3B). In contrast, it accelerates the relaxation of basal cardiomyocytes following β_2AR stimulation (Figure 3B).

MCD treatment is a coarse method of removing cellular caveolae. To confirm our findings, we utilized apical and basal cells from mice in which Cav3 was knocked down in cardiac myocytes. This removed most cellular caveolae from the Cav3 knockout (KO) basal cardiomyocytes (Figure S3B). After mice had been treated with tamoxifen to remove Cav3 gene expression, both apical and basal cells became equally responsive to β_2AR stimulation (Figure 3C). Like MCD treatment, following Cav3 knockdown, apical cells did not increase their relaxation velocity in response to β_2AR stimulation (Figure 3D). Data showing the effectiveness of the tamoxifen-inducible conditional promoter are presented in Figures 3E and 3F. Faithful knockdown of Cav3 mRNA production is demonstrated in both apical and basal cells (Figure S3C). Neither KO of Cav3KO or removal of cholesterol from cell membranes appears to affect T-tubular density or regularity (Figures S4C–S4F).

β_2AR-cAMP Responses in Both Plasma Membrane and RII Nanodomains Are Greater in Rat Apical Cardiomyocytes

We measured local cAMP production to assess the relative level of compartmentation in different subcellular nanodomains upon generation of cAMP following β_2AR stimulation. Apical and basal cardiomyocytes were transfected with either a plasma membrane-targeted (pmEPAC2) or a PKA_RII nanodomain-targeted sensor (RII_EPAC). cAMP production at the plasma membrane following β_2AR stimulation was found to be greater in apical than in basal cardiomyocytes (Figures S5A and S5B). Basal cells show smaller but substantial cAMP production in the membrane nanodomains. This difference becomes more pronounced when

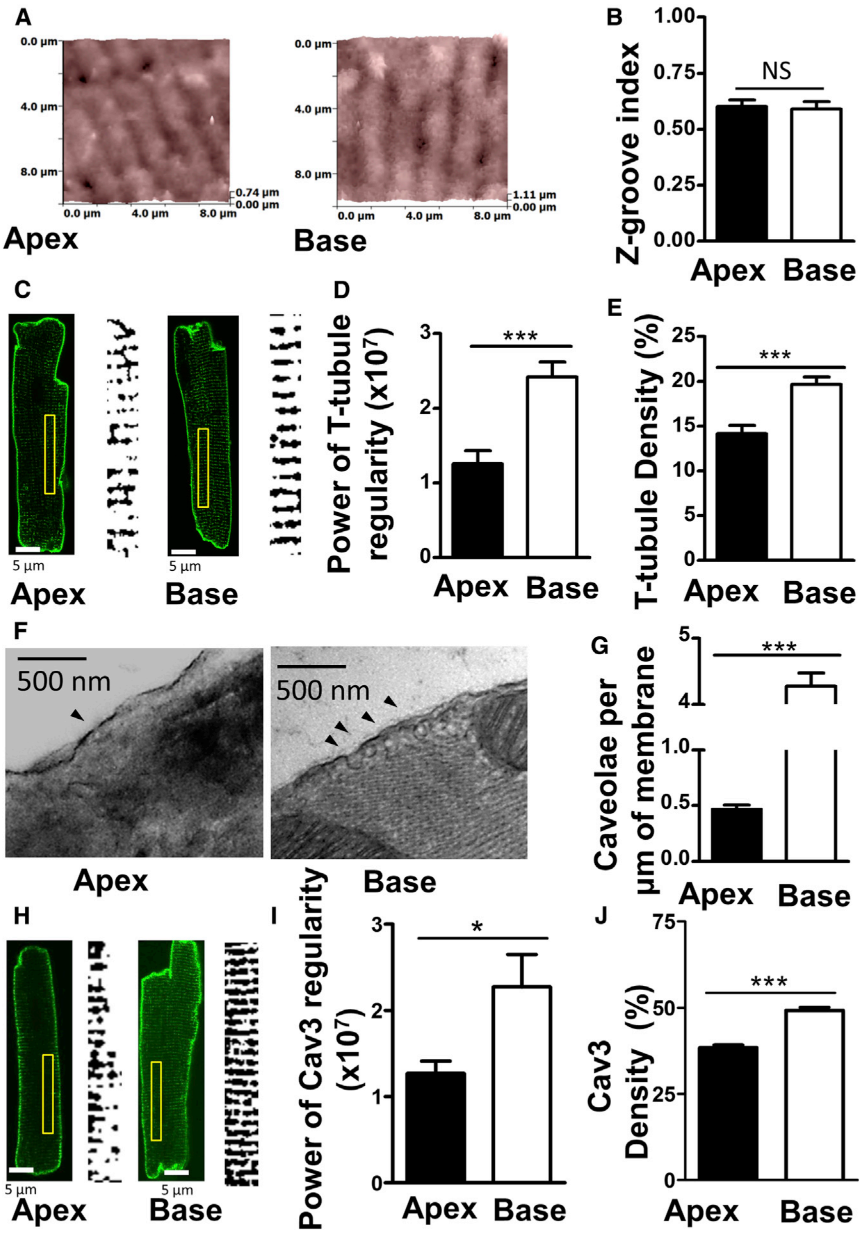

Figure 2. Intracellular Organization of Apical and Basal Myocytes from Rats

(A) Representative SICM images of the surface topography of apical and basal cardiomyocytes.

(B) Quantification of the degree of myocyte surface structural organization by Z-groove index (apical/basal, N, n = (5) 45/44, NS, unpaired t test).

(C) Tubular membrane organization of di-8AN-NEPPS-stained apical and basal cardiomyocytes. Insets show the areas chosen to make binary images for analysis.

(D and E) Quantification of the relative regularity (D) and density (E) of T-tubules (N, n = (3) 15, $p < 0.001$).

(F) Examples of electron micrographs showing plasma membrane cross-sections of apical and basal myocytes; N = 10.

(G) Quantification of the density of caveolae in apical and basal cardiomyocytes from EM images. $p < 0.001$, unpaired t test.

(H) Examples of immunostaining of Cav3 in apical and basal myocytes.

(I) Morphometric quantification of the regularity of Cav3 staining (from immunostaining micrographs).

(J) Quantification of the density of Cav3 staining. N, n = (3) 12/13, NS, unpaired t test.

Error bars indicate SEM.

the response is normalized to total cAMP output. A significantly larger difference in cAMP response following β_2AR stimulation was measured in apical cells by the RII_EPAC sensor than in basal cardiomyocytes (Figure S5C). Normalization of β_2AR-dependent cAMP response to the total cAMP output clearly shows a higher sensitivity of apical cells in comparison with basal cells in the RII domain (Figure S5D).

The Removal of Caveolae Increases the Access of β_2AR-cAMP to Specific Subcellular Nanodomains in Rat Cardiomyocytes

We wanted to find out whether the changes in contractile response we observed following the removal of caveolae were due to the altered cAMP access to different nanodomains. Treatment with MCD did not change β_2AR-dependent cAMP responses measurable in the cytosol of both apical and basal cardiomyocytes (Figures 4A and 4D). Moreover, in apical cells, no significant change is seen in both RII_PKA and membrane compartments (Figures 4B and 4D). In basal cardiomyocytes, a smaller cAMP response was observed following β_2AR stimulation in either RII or membrane domains (Figures 4C and 4D). However, pre-treating the basal cardiomyocytes with MCD leads to the appearance of a cAMP response in both RII domains (Figure 4B) and at the plasma membrane (Figure 4C). Correction of the β_2AR-dependent cAMP response to total cAMP also shows that MCD pre-treatment increases the β_2AR response of basal cardiomyocytes in both RII (Figure 4B) and membrane (Figure 4C) domains, equilibrating the apical and basal responses.

Inhibition of PDE4 Using Rolipram Enhances β_2AR-cAMP Responses and Equilibrates Rat Apical and Basal Cell Contractile Responses to β_2AR Stimulation

One way of restricting the production of cAMP in nanodomains is to regulate how fast it is inactivated by PDEs. We used inhibitors of three PDEs known to be expressed in cardiac myocytes to assess the role of each isoform in the control of cAMP within apical and basal cells using a cytosolic FRET sensor. PDE2 and

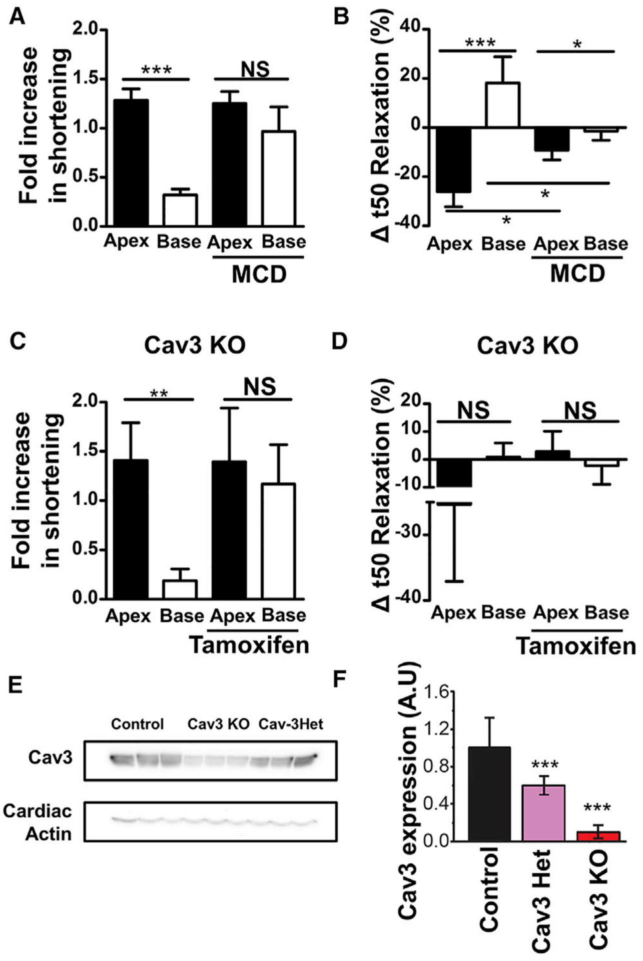

Figure 3. Role of Caveolae in Restraining Contraction of Basal Myocytes following β_2AR Stimulation

(A) Effect of MCD (1–2 mM) pre-treatment on the shortening responses of apical and basal cardiomyocytes from rats following β_2AR stimulation with 1 μM ISO + 300 nM CGP. N, n = (3) 6/6, $p < 0.001$, apex versus base, unpaired t test.

(B) Effect of MCD pre-treatment on the relative change in relaxation speed of rat apical and basal cardiomyocytes following β_2AR stimulation. N, n = (3) 6/6, $p < 0.05$, $p < 0.001$, unpaired t test.

(C) The influence of genetic KO of Cav3 on the shortening responses of apical and basal cardiomyocytes from mice following β_2AR stimulation. N, n = (3) 6/6, $p < 0.01$, unpaired t test.

(D) Effects of conditional genetic KO of Cav3 on the relative change in relaxation speed of apical and basal cardiomyocytes following β_2AR stimulation. N, n = (4) 9/10, NS, unpaired t test.

(E) Western blot analysis of Cav3 levels in cardiac myocytes isolated following tamoxifen treatment of cav3flox/flox/Mer-αMHC−MerCre+/− mice.

(F) Quantification of the amount of Cav3 on the blots in (E) by densitometry.
Error bars indicate SEM. See also Figures S2–S4.

PDE3 activity was not significantly different between the apex and base (Figure S6A). Inhibition of PDE4 with rolipram in apical and basal cells resulted in both cell types displaying increased contractility at baseline (Figure S2C) compared with control values (Figure S2A). However, inhibiting PDE4 alone is not enough to provide maximum contractile output at baseline because, upon β_2AR stimulation, increased contraction was observed in both apical and basal cells (Figure S2C); this effect is similar to MCD treatment (Figure S2B). PDE4 is considered to predominate in removing cAMP from the plasma membrane and PKA compartments in rodent cardiomyocytes (Richter et al., 2011). We sought to find out whether the observed decrease (as reported in previous figures) in localized cAMP responses to β_2AR stimulation in basal cells is a result of increased PDE4 activity. We used either the global sensor cEPAC2-camps or the localized sensor RII_EPAC. Perfusion of rolipram in apical cells after β_2AR stimulation results in little further increase in cAMP in either the cytosol or RII domains; however, basal cells demonstrate an increased β_2AR-cAMP response under these conditions in both the cytosol and RII domains (Figures 5A and 5B). Normalized values for the β_2AR-dependent contractile responses (Figure 5C) following PDE4 inhibition are equilibrated in apical and basal cells, which is reminiscent of both MCD treatment (Figure 3A) and Cav3 removal (Figure 3C). Without rolipram, only the apical cells' relaxation velocity significantly decreased (time to relaxation to 50% of baseline length [R50] increased) after β_2AR stimulation. In the presence of rolipram, basal cells increased in relaxation velocity after β_2AR stimulation, but this did not reach statistical significance (Figure 5D).

PDE4 Localization in Caveolae Contributes to Apical/Basal Differences in Rat Cells

To assess how quickly the cAMP produced following β_2AR stimulation is depleted in the RII compartment, we looked at the FRET ratio over time following a rapid washout of ISO. The cAMP levels remained elevated longer after stimulation in apical versus basal cells (Figures S6F and S6I). Treatment of both cell types with rolipram (Figures S6G and S6J) or MCD (Figures S6H and S6K) removed this difference and equilibrated the relative persistence of cAMP in these nanodomains. In addition to functional assays in isolated cells, we performed a biochemical assay of the PDE4 activity on isolated caveolin-rich membrane fractions of myocytes. Higher PDE4 activity was observed in basal cells compared with apical cells (Figure 5E). Moreover, the total PDE activity is also higher in the caveola-rich membrane fraction of basal cardiomyocytes compared with apical cells (Figure 5E). A significantly greater amount of PDE4B but not PDE4D (although there is a strong tendency) is apparent in basal buoyant fractions in comparison with those isolated from the apex (Figures S6B and S6E).

β_2AR-Dependent Production of cAMP Is Greater in the Apex of a Perfused Mouse Heart Than in the Base

Having observed the differences of β_2AR-cAMP signaling compartmentation in apical and basal cardiomyocytes from rat and mouse, we wanted to see whether these differences exist at the level of the whole heart. We monitored cAMP production in Langendorff-perfused hearts isolated from pmEPAC2-camps transgenic mice following baseline measurements and perfusion with CGP and with treatment with ISO to produce a β_2AR-stimulated cAMP response. After a period of washout, the PDE4 blocker rolipram, along with CGP and ISO, was perfused into the heart to reveal the relative control of cAMP by this PDE subtype following β_2AR stimulation. Greater FRET responses were

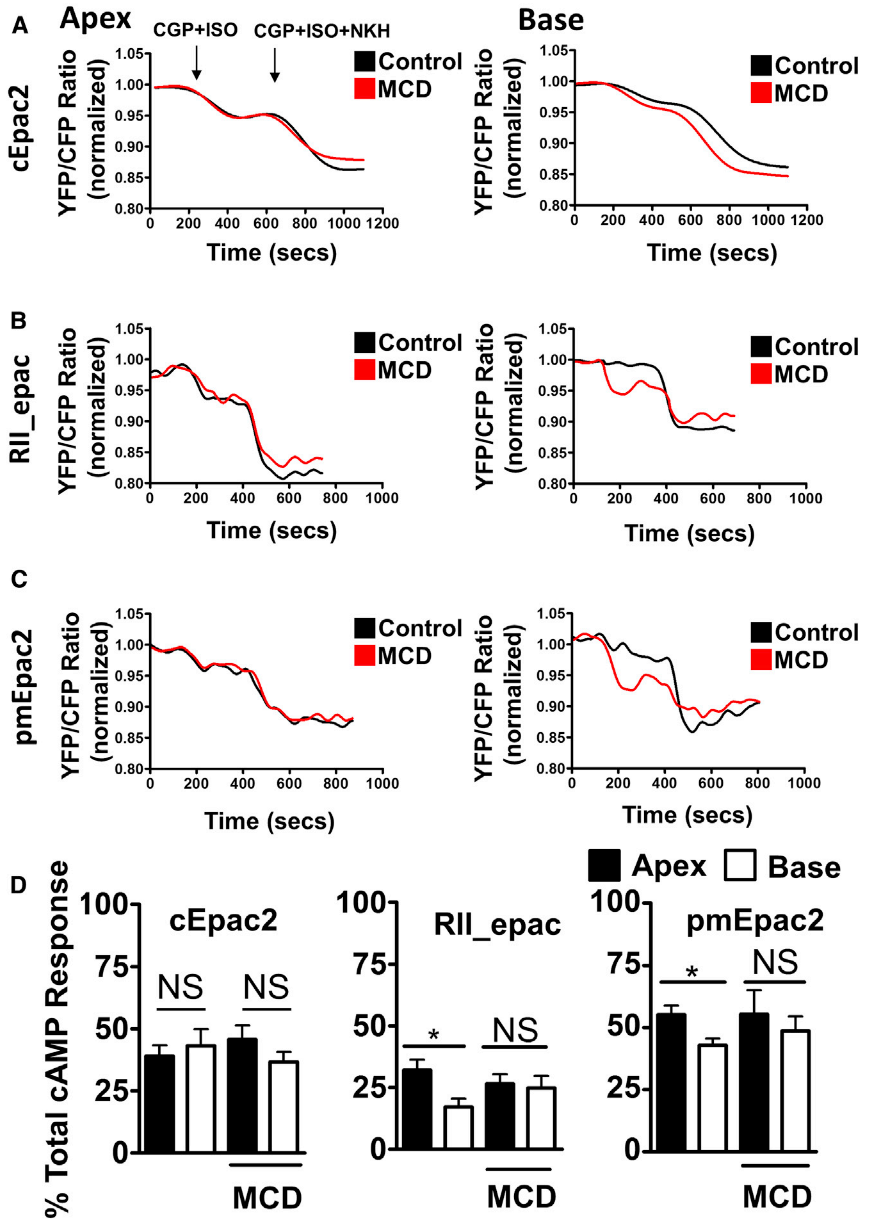

Figure 4. Role of Caveolae/Cholesterol in Compartmentation of β_2AR Signaling in the Apex and Base

(A–C) Representative traces showing rat apical and basal cell cAMP responses with or without MCD (1–2 mM) pre-treatment following β_2AR stimulation with 100 nM ISO + 100 nM CGP, as measured with a cytosolic (cEPAC2) FRET biosensor (A), in the PKA_RII compartment (RII_EPAC) (B), and in the plasma membrane compartment (pmEPAC2) (C).

(D) Quantification of cAMP responses corrected to total cAMP output. Apex/base cEPAC2, N, n = (5) 22/14, (5) 10/7; RII_EPAC, N, n = (5) 20/20, 11/15; and pmEPAC2, N, n = (5) 16/18, 15/15; unpaired t test; $^*p < 0.05$.

Error bars indicate SEM. See also Figures S4 and S5.

measured at the apex in comparison with the base (Figures 6A and 6B) following CGP and ISO stimulation, suggesting a greater β_2AR response in the apex. Following washout, FRET signals rapidly dissipated in both the apical and basal regions.

Perfusion with CGP+ISO+rolipram then caused a large FRET response in both regions. Following image analysis, a significant difference in β_2AR-stimulated cAMP responses can be observed between the apex and the base (Figure 6C). A significant difference (paired measurement) was observed between CGP+ISO and CGP+ISO+rolipram (ROLI) steps in the basal segment but not the apical one, suggesting a comparatively greater role for PDE4-mediated cAMP control in this region (Figure 6C). Overall, larger responses were observed at both stages within the apical segment. We repeated this experiment to examine the effect of epinephrine, a more physiologically relevant ligand, delivery into intact perfused pmEpac1 hearts. A significantly greater amount of cAMP is produced in the apical segment in comparison with the basal, after application of this agent (Figure S7).

DISCUSSION

Different regional responses to stress and high catecholamine levels are observed in human hearts after stressful experiences and in animal models of stress or epinephrine infusion. The studies here demonstrate that contractile responses of cardiomyocytes from separate myocardial regions differ following β_2AR stimulation. The presence of a cAMP response in the cytosol following β_2AR stimulation does not necessarily result in a contractile response. We present data that suggest that the characteristics of left ventricular cardiomyocyte responsiveness to β_2AR stimulation are linked to regional origin. The concept central to our hypothesis is that cAMP must be conducted from its region of production (the plasma membrane) to cAMP-dependent effector molecules (the RII domains of PKA). These are spatially separate, so this process may involve transit through a region both separate from production and/or not involved in effector control (the cytosol). Our data demonstrate that less β_2AR-cAMP is detected in the plasma membrane and PKA-RII nanodomains of basal cardiomyocytes following β_2AR stimulation compared with apical cardiomyocytes. We propose that regional differences are the result of local heterogeneity in effective cAMP generation and removal. These processes are controlled to a large degree by compartmentation of β_2AR-cAMP by membrane organization and PDE4. cAMP compartmentation appears to be reliant on the

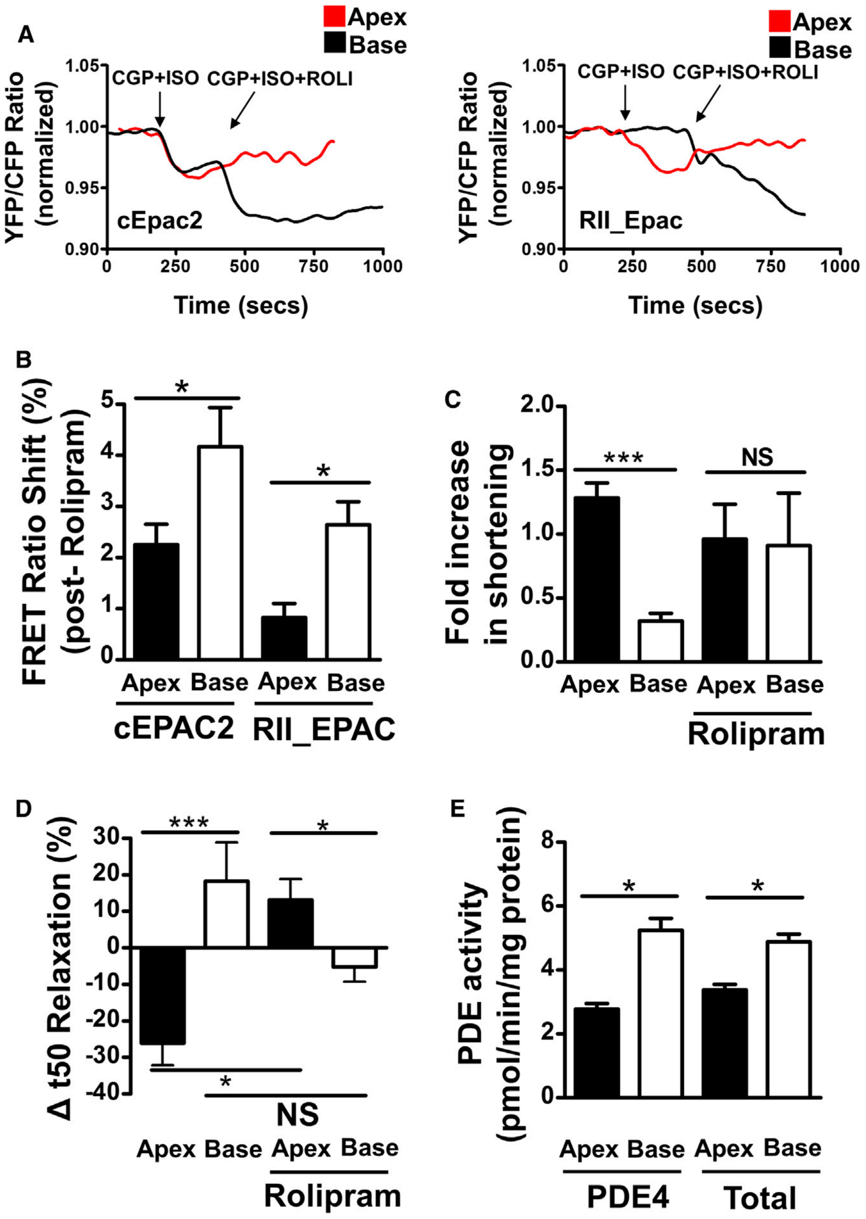

Figure 5. Investigating the Role of PDE4 in Compartmentation of β_2AR Signaling in the Apex and Base by Inhibition with ROLI

(A) Representative trace showing the FRET responses of rat apical and basal cells following the stimulation of β_2AR (100 nM ISO + 100 nM CGP) and subsequent inhibition of PDE4 by ROLI (5 μM) infusion, measured in cytosolic and RII_PKA compartments.

(B) Quantification of the relative increase in FRET response following infusion of ROLI. N, n = (6)21/22, n = 13/13, unpaired t test, p < 0.05.

(C) Effect of ROLI pre-treatment on the shortening responses of apical and basal cardiomyocytes following β_2AR stimulation. N, n = (3)5, unpaired t test, ***p < 0.001.

(D) Effect of ROLI pre-treatment on the relative change in relaxation speed of apical and basal cardiomyocytes following β_2AR stimulation. Unpaired t test, *p < 0.05, ***p < 0.001.

(E) Specific PDE4 and total PDE activity in apical and basal cell caveolar membrane preparations. N, n = (6)6, unpaired t test, *p < 0.05.

Error bars indicate SEM. See also Figures S2 and S6.

structural organization of the cardiomyocyte, specifically the number of caveolae and the density and regularity of t-tubular structures. As a result of this work, we can propose the schematic illustrated in Figure 7.

In the apical myocardium, agonists of β_2AR stimulate receptors that are not fully compartmentalized because of the reduction in membrane complexity. As a result, the cAMP is less well controlled by the hydrolytic activity of PDE4. As a result, persistent β_2AR-cAMP penetrates PKA-RII compartments, and molecular actors within the excitation-contraction coupling system are more effectively phosphorylated. In response, apical cells increase their contractility to a greater degree than basal cardiomyocytes following β_2AR stimulation. Perhaps even more interestingly, we note that basal cardiomyocytes seem to be actively concentrating PDE4B in their caveolae, which is likely to contribute to the greater compartmentation of cAMP alongside the enhancement of membrane complexity.

Our study shows a clear relationship between the persistence of cAMP within the RII domains and the physiological response of cardiomyocytes to β_2AR stimulation. Pharmacological interventions that increase cAMP in these domains in comparison with a control also appeared to cause increased inotropic and lusitropic responses, in basal cells in particular. This finding is similar to that found in the work of Macdougall et al. (2012). The selective β_2AR agonist zinterol elicited cAMP responses in the cardiomyocyte cytosol (measured by the cEPAC2 sensor) but not PKA-RII compartments. These data, from cells isolated as a homogenate from the entire left ventricle, bears similarities with cells isolated from the basal myocardium in our study. On the basis of these data, we hypothesize that the apical myocardium represents a discrete portion of the rat heart from a pharmacological perspective. The question of whether the β_2AR can control excitation-contraction coupling has been investigated in cardiac cells from different organisms of differing levels of maturity and via different metrics (Balijepalli et al., 2006; Kuschel et al., 1999; Rybin et al., 2003). Studies have assessed the effect of β_2AR on cell contractility. The findings of these publications have been somewhat divergent, with some reporting a role for β_2AR in the control of adult cardiomyocyte contractility or members of the excitation-contraction coupling

OPEN ACCESS
CellPress

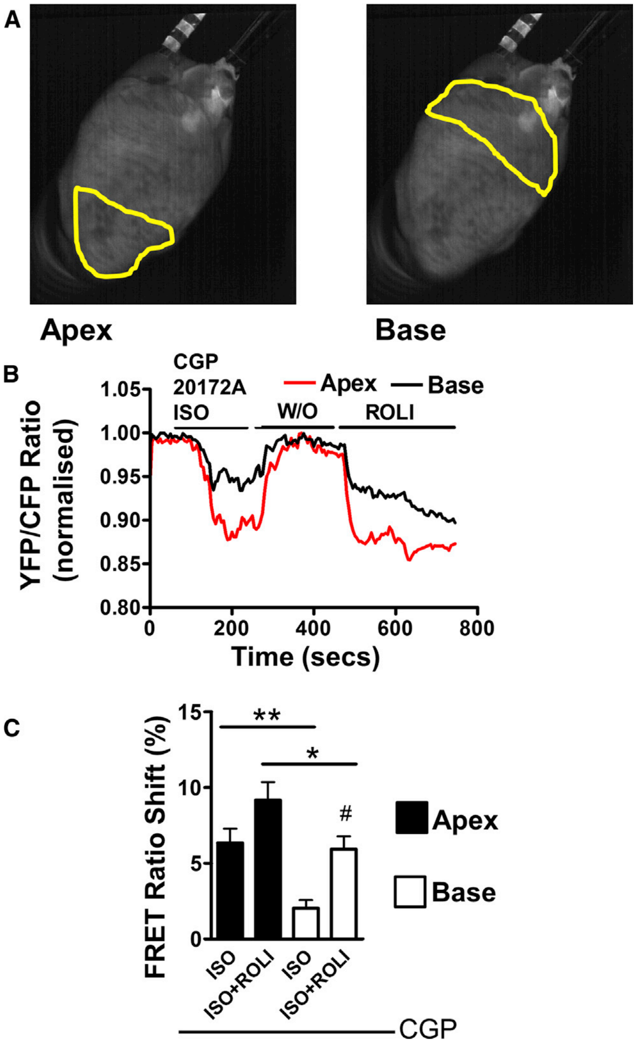

Figure 6. Imaging Regional Differences in β_2AR-cAMP Responses in a Langendorff-Perfused Mouse Heart

(A) Images demonstrating the selection of regions for FRET analysis (these are broadly representative of the regions dissected for cells in other studies) to allow assessment of FRET responses. Images show the yellow fluorescence channel (FRET acceptor).

(B) Representative traces presenting the responses of the apical and basal portions of the myocardium under different pharmacologic stimuli.

(C) Quantification of FRET responses to β_2AR stimulation (100 nM ISO + 100 nM CGP) and ROLI (5 μM) from the apical and basal sectors. N, n = (8) 8, $p < 0.05$, unpaired t test; #$p < 0.05$, paired t test, CGP+ISO versus CGP+ISO+ROLI steps.

Error bars indicate SEM. See also Figure S7.

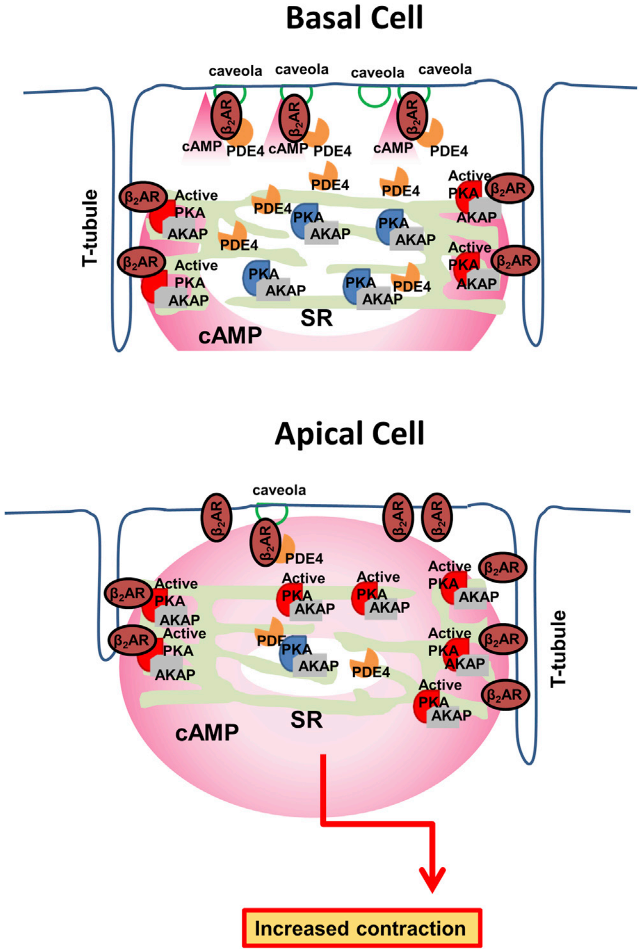

Figure 7. Simplified Depiction of the Differences in β_2AR Nanodomains in Apical and Basal Cardiac Myocytes

Top: the scenario within relatively well organized basal cardiomyocytes. A high degree of membrane organization in basal cardiomyocytes allows tight control of the cAMP level because of enhanced efficacy of PDE4-mediated cAMP hydrolysis. As a result, the cAMP produced by β_2AR stimulation cannot modulate cell contractility to a great extent. Bottom: the scenario of less organized apical cardiomyocytes. Apical cardiomyocyte membranes lack extensive nanodomain compartmentation, and, as a result, β_2AR-stimulated cAMP is able to reach deeper from the membrane and increases cellular contractility via PKA-mediated control of the excitation contraction (EC) coupling machinery.

machinery. Equally, earlier studies employed either pertussis toxin (PTX) or 3-isobutyl-1-methylxanthine (IBMX) (Balijepalli et al., 2006; Chen-Izu et al., 2000; Kuschel et al., 1999; Rybin et al., 2003). We suggest that the loss of β_2AR control/compartmentation brought about by these agents would also make the cells less reflective of the situation *in vivo*. We propose that latent heterogeneity within ventricular cardiomyocytes may have contributed to the differing findings of these studies as well as the occasional employment of either IBMX or PTX.

The necessity for the differences between apical and basal cardiomyocyte populations within the myocardium is unclear. However, computational modeling has revealed that fiber strain and external work are lowest at the apical epicardium and highest at the mid-left ventricular endocardium and basal epicardium (Usyk et al., 2000). For βAR stimulation to optimally increase cardiac output, it can be assumed that the regional functional properties need to be differentially enhanced. Thus, the differences in sympathetic functional responses we report may be necessary to selectively enhance apical function to avoid left ventricular outflow track obstruction. This may especially be true of situations where there are periods of elevated sympathetic activation; for example, exercise or emotional stress.

The apical ventricular wall is thinner than the basal part; this provides a conical structure that efficiently propels blood. The anatomy of the apical myocardium coupled with enhanced adrenergic responsiveness makes it an ideal reservoir of contractile force that can be deployed in stressful situations. Stretching cardiac tissue or cells has been demonstrated to decrease conduction velocity in pathological scenarios and in health (Mills et al., 2011). Membrane caveolae are recruited and removed from the plasma membrane by stretch (Kohl et al., 2003). The presence of extra caveolar membranes and the subsequent tighter control of β_2AR-dependent cAMP production in the base may be a consequence of this important mechanism. The lower amount of caveolae in the apical cells may reduce membrane capacitance, preventing conduction slowing (Kohl et al., 2003).

There are also notable gradients in innervation within the myocardium. The basal myocardium displays a greater degree of innervation than the apical segment (Kawano et al., 2003). One may hypothesize that the presence of a greater density of nerves may inversely drive the expression and control of β_2ARs. It has been reported that innervation reduces the number of caveolae in neonatal cardiomyocytes in the proximity of the synapse (Shcherbakova et al., 2007). This is somewhat countervailing the findings of this study. The control of caveolae may be more dynamic than these studies have illustrated and different within the adult myocardium. The differences in the control of β_2AR may be modified in heart disease, such as Takotsubo syndrome, where regional differences in contractile function following stress or exogenous catecholamine administration are overtly present. Future studies should investigate to what extent the regional heterogeneity described in this study is the result of differences in the physical stresses present in different organ microenvironments. In summary, we present a case of cellular micro-domains building tissue/organ-level differences in physiology, which may be important for the control of physiology of many other organs.

EXPERIMENTAL PROCEDURES

Animals

Animal experiments met the criteria of Imperial College London and Animals in Scientific Procedures Act (ASPA) 1986 as well as the 2010/63/EU Directive. Apical and basal cardiomyocytes were isolated from the left ventricles of male Sprague Dawley rats (200–500 g), male/female Cav3KO mice (20–30 g), or male/female β_2AR KO mice (20–30 g) (Devic et al., 2001) by a Langendorff perfusion method as described previously (Nikolaev et al., 2010, Paur et al., 2012; Figure S1). After brief enzymatic perfusion, the hearts were cut down from the Langendorff system. The atria and right ventricle were excised. The remaining left ventricle was divided into three, and the middle section was discarded. The basal and apical sections were disrupted and shaken separately to yield isolate myocytes from separate regions. The cells from the two populations survived with a rod-shaped morphology beyond 72 hr.

Cav3 conditional KO mice were generated by crossing a mouse line with loxP-flanked exon 2 of Cav3 with an alpha-myosin heavy chain proto-oncogene tyrosine-protein kinase MER Cre recombinase proto-oncogene tyrosine-protein kinase MER (αMHC-MERCreMER) (Sohal et al., 2001) mouse producing a tamoxifen-inducible, cardiac-specific Cav3 KO mouse (Markandeya et al., 2015). Mice were given an intraperitoneal injection of 1 mg tamoxifen/day for 3 consecutive days and sacrificed 21 days post-tamoxifen treatment, and cells were isolated as described above. Hearts from male and female pmEpac2 mice were used for *ex vivo* FRET measurements.

IonOptix-Based Measurements of Cell Contractility

The fractional shortening of cardiomyocytes was assessed on the day of isolation by the IonOptix method (Westwood, MA, US). Cells were perfused with Krebs-Henseleit (37°C, 95%/5% O_2:CO_2) paced at 0.5 Hz (rat) or 1 Hz (mouse). The cells of both species were paced (50 V/pulse width, 2 ms). After a baseline period, β_1 blockade was achieved by perfusing cells with CGP (300 nM) for 10 min (or β_2 blockade with ICI). Subsequently, ISO was used to stimulate cells' β_2AR (1 μM). Following analysis, the shift in fractional shortening (%FS) and the time to R50 was assessed from ten representative peaks from each phase of pharmacologic treatment.

Measurement of Intracellular cAMP Responses by FRET Microscopy

Isolated cardiomyocytes were plated on laminin-coated coverslips and cultured for 48–56 hr in M199 medium and transfected with FRET constructs by adenoviral delivery. The cEPAC2-cAMPs and pmEPAC2 adenoviruses were a kind gift from Prof. Martin Lohse (University of Würzburg), and the RII_EPAC sensor was a gift from Prof. Manuela Zaccolo (University of Oxford) (Stangherlin et al., 2011). Cells were analyzed between 44 and 56 hr post-transfection. β_1AR was blocked by CGP (100 nM), and β_2AR was then stimulated with ISO (100 nM). Finally, total adenyl cyclase (AC) activity was elicited by NKH477 (5 μM) stimulation to allow correction. Variations of this method were performed. The PDE4 blocker ROLI (10 μM) was substituted for NKH477 following β_2AR stimulation to reveal PDE4 tone in cardiomyocytes. Erythro-9-(2-hydroxy-3-nonyl)adenine (EHNA) (10 μM) was used to probe the function of PDE2, and milrinone (1 μM) was used to assess the role of PDE3. IBMX-sensitive PDEs were probed by perfusion with IBMX (100 μM). Rapidly washing ISO off following β_2AR stimulation, with no further stimulation, allowed assessment of the kinetics of cAMP hydrolysis/removal within cells. Images of CFP and yellow fluorescent protein (YFP) fluorescence were acquired using a digital camera and optical microscopy system. The intensities of the images were ratioed, and a bleedthrough factor of 0.956 was subtracted and normalized to baseline. Measurements of FRET shifts were then acquired from curves in a number of ways depending on the property or methodology of interest.

Measurement of Surface Organization by SICM

SICM measurements were performed as described previously (Nikolaev et al., 2010). The cell surface was scanned to allow visualization of T-tubules via the scanning pipette. Calculation of the Z-groove index was produced by measuring the length of Z-grooves from single SICM images and dividing this by the hypothesized maximal Z-groove length in a 10 × 10 μm scan (50 μm).

Measurement of FRET Responses in Langendorff-Perfused Hearts

FRET-based measurements were performed in Langendorff-perfused hearts harvested from pmEPAC2-camps transgenic mice as described previously (Wachten et al., 2010). After measurement of baseline activity, hearts were perfused with epinephrine (100 nM) or CGP (100 nM) and then ISO (100 nM) ± ROLI (5 μM) (both from Sigma-Aldrich, St. Louis, MO, USA). We used an imaging system created around a Leica M165FC (Leica Microsystems, Wetzlar, Germany) stereomicroscope (for Langendorff perfusion) to measure FRET responses. The cAMP sensor was excited with a 440-nm light emitting diode (LED) (pE-100, CoolLED, Andover, UK) source. Emission light was split into donor and acceptor channels using a DV2 DualView beamsplitter apparatus equipped with a 565DCXR dichroic mirror and D480/30 and D535/40 emission filters (Photometrics, Tucson, AZ, USA). Images were taken using an optiMOS camera (Photometrics, Tucson, AZ, USA) with MicroManager 1.4 open source imaging software and analyzed by ImageJ (NIH, USA) with in-house plugins. Raw data were corrected offline for the bleedthrough factor of the donor into the acceptor channel (Nikolaev et al., 2010).

Microscopic Techniques: Confocal and Electron Microscopy

Detailed descriptions of our methodology can be found in the Supplemental Experimental Procedures.

PDE Activity Assay

A full description of the methodology can be found in the Supplemental Experimental Procedures. We used the method of Head et al. (2005) to allow

preparation of caveolar and non-caveolar membrane fractions. Our PDE assay was an adaptation of a previous method (Thompson and Appleman, 1971) to determine the cAMP hydrolysis capacity of tissue preparations.

Statistical Analysis

Data were tested for normality (Kolmogorov-Smirnov test). Where two unrelated populations were analyzed, statistical difference was determined using an unpaired (two-tailed) Student's t test. Where more than two unrelated populations were analyzed, one-way ANOVA with a Bonferroni post-test was used. This was performed using GraphPad 4.0 software. In the figure legends, N refers to the number of preparations, and n refers to the number of measurements.

Data Availability

The data presented in this study are available from the authors upon request.

SUPPLEMENTAL INFORMATION

Supplemental Information includes Supplemental Experimental Procedures and seven figures and can be found with this article online at https://doi.org/10.1016/j.celrep.2018.03.053.

ACKNOWLEDGMENTS

We would like to acknowledge Peter O'Gara for performing cardiomyocyte isolation. We thank the NHS Brompton EM facility for their assistance with the quantification of caveolar domains presented in this study. We would also like to thank Professor Kristina Lorenz for her gift of β_2ARKO mice and her laboratory members Bettina Tanitha Baumgarten and Jonas Bodmann for their assistance with β_2ARKO studies. This work was supported by the British Heart Foundation (JG12/18/30088, RG/17/13/33173, MZ-RG/12/3/29423, and PG/15/5/31110), the Medical Research Council (JG+PW MR/L006855/1), and the NIH (R01HL078878 to T.J.K. and R.C.B. and P50GM094503 to T.J.K.). V.O.N. received funding from the Gertraud und Heinz Rose Foundation.

AUTHOR CONTRIBUTIONS

P.T.W. conceived the research question, designed the research, performed experiments, and authored the manuscript. I.D., L.M.P.K., N.I.B., A.A.-L., S.S., C.L., and N.K.B. performed experiments. R.K.P. designed and performed experiments. G.F. provided funding support. M.Z. contributed essential reagents and co-authored the manuscript. T.J.K. and R.C.B. contributed the animal model and co-authored the manuscript. A.R.L. and S.E.H. co-authored the manuscript and designed experiments. V.O.N. contributed essential reagents, co-authored the manuscript, and designed and performed experiments. J.G. oversaw the entirety of the research, conceived the research question, designed experiments, and co-authored the manuscript.

DECLARATION OF INTERESTS

The authors declare no competing interests.

Received: May 16, 2017
Revised: February 2, 2018
Accepted: March 13, 2018
Published: April 10, 2018

REFERENCES

Balijepalli, R.C., Foell, J.D., Hall, D.D., Hell, J.W., and Kamp, T.J. (2006). Localization of cardiac L-type Ca(2+) channels to a caveolar macromolecular signaling complex is required for β(2)-adrenergic regulation. Proc. Natl. Acad. Sci. USA *103*, 7500–7505.

Bers, D.M. (2002). Cardiac excitation-contraction coupling. Nature *415*, 198–205.

Buxton, I.L., and Brunton, L.L. (1983). Compartments of cyclic AMP and protein kinase in mammalian cardiomyocytes. J. Biol. Chem. *258*, 10233–10239.

Carr, D.W., Hausken, Z.E., Fraser, I.D., Stofko-Hahn, R.E., and Scott, J.D. (1992). Association of the type II cAMP-dependent protein kinase with a human thyroid RII-anchoring protein. Cloning and characterization of the RII-binding domain. J. Biol. Chem. *267*, 13376–13382.

Chen-Izu, Y., Xiao, R.P., Izu, L.T., Cheng, H., Kuschel, M., Spurgeon, H., and Lakatta, E.G. (2000). G(i)-dependent localization of beta(2)-adrenergic receptor signaling to L-type Ca(2+) channels. Biophys. J. *79*, 2547–2556.

Devic, E., Xiang, Y., Gould, D., and Kobilka, B. (2001). β-adrenergic receptor subtype-specific signaling in cardiac myocytes from β(1) and β(2) adrenoceptor knockout mice. Mol. Pharmacol. *60*, 577–583.

Di Benedetto, G., Zoccarato, A., Lissandron, V., Terrin, A., Li, X., Houslay, M.D., Baillie, G.S., and Zaccolo, M. (2008). Protein kinase A type I and type II define distinct intracellular signaling compartments. Circ. Res. *103*, 836–844.

Head, B.P., Patel, H.H., Roth, D.M., Lai, N.C., Niesman, I.R., Farquhar, M.G., and Insel, P.A. (2005). G-protein-coupled receptor signaling components localize in both sarcolemmal and intracellular caveolin-3-associated microdomains in adult cardiac myocytes. J. Biol. Chem. *280*, 31036–31044.

Heather, L.C., Catchpole, A.F., Stuckey, D.J., Cole, M.A., Carr, C.A., and Clarke, K. (2009). Isoproterenol induces in vivo functional and metabolic abnormalities: similar to those found in the infarcted rat heart. J. Physiol. Pharmacol. *60*, 31–39.

Kawano, H., Okada, R., and Yano, K. (2003). Histological study on the distribution of autonomic nerves in the human heart. Heart Vessels *18*, 32–39.

Kohl, P., Cooper, P.J., and Holloway, H. (2003). Effects of acute ventricular volume manipulation on in situ cardiomyocyte cell membrane configuration. Prog. Biophys. Mol. Biol. *82*, 221–227.

Kuschel, M., Zhou, Y.-Y., Spurgeon, H.A., Bartel, S., Karczewski, P., Zhang, S.-J., Krause, E.-G., Lakatta, E.G., and Xiao, R.-P. (1999). β2-adrenergic cAMP signaling is uncoupled from phosphorylation of cytoplasmic proteins in canine heart. Circulation *99*, 2458–2465.

Lathers, C.M., Levin, R.M., and Spivey, W.H. (1986). Regional distribution of myocardial β-adrenoceptors in the cat. Eur. J. Pharmacol. *130*, 111–117.

Liu, R., Wang, D., Shi, Q., Fu, Q., Hizon, S., and Xiang, Y.K. (2012). Palmitoylation regulates intracellular trafficking of β_2 adrenergic receptor/arrestin/phosphodiesterase 4D complexes in cardiomyocytes. PLoS ONE *7*, e42658.

Lunkenheimer, P.P., Redmann, K., Kling, N., Jiang, X., Rothaus, K., Cryer, C.W., Wübbeling, F., Niederer, P., Heitz, P.U., Ho, S.Y., and Anderson, R.H. (2006). Three-dimensional architecture of the left ventricular myocardium. Anat. Rec. A Discov. Mol. Cell. Evol. Biol. *288*, 565–578.

Lyon, A.R., Bossone, E., Schneider, B., Sechtem, U., Citro, R., Underwood, S.R., Sheppard, M.N., Figtree, G.A., Parodi, G., Akashi, Y.J., et al. (2016). Current state of knowledge on Takotsubo syndrome: a Position Statement from the Taskforce on Takotsubo Syndrome of the Heart Failure Association of the European Society of Cardiology. Eur. J. Heart Fail. *18*, 8–27.

Macdougall, D.A., Agarwal, S.R., Stopford, E.A., Chu, H., Collins, J.A., Longster, A.L., Colyer, J., Harvey, R.D., and Calaghan, S. (2012). Caveolae compartmentalise β2-adrenoceptor signals by curtailing cAMP production and maintaining phosphatase activity in the sarcoplasmic reticulum of the adult ventricular myocyte. J. Mol. Cell. Cardiol. *52*, 388–400.

Mantravadi, R., Gabris, B., Liu, T., Choi, B.-R., de Groat, W.C., Ng, G.A., and Salama, G. (2007). Autonomic nerve stimulation reverses ventricular repolarization sequence in rabbit hearts. Circ. Res. *100*, e72–e80.

Markandeya, Y.S., Feng, L., Ramchandran, V., Vaidyanathan, R., Best, J., Lea, M.L., Keefe, A., Kalscheur, M.M., O'Hara, T., Eckhardt, L.L., et al. (2015). Abstract 19749: Cardiac-specific Deletion of Caveolin-3 Delays Repolarization and Increases Susceptibility to Ventricular Arrhythmia. Circulation *132*, A19749.

Mills, R.W., Wright, A.T., Narayan, S.M., and McCulloch, A.D. (2011). The effects of wall stretch on ventricular conduction and refractoriness in the whole heart. Card. Mechano-Electric Coupling Arrhythmias, P. Kohl, F. Sachs, and M.R. Franz, eds. (Oxford: Oxford Univ. Press), pp. 180–186.

Mori, H., Ishikawa, S., Kojima, S., Hayashi, J., Watanabe, Y., Hoffman, J.I.E., and Okino, H. (1993). Increased responsiveness of left ventricular apical myocardium to adrenergic stimuli. Cardiovasc. Res. *27*, 192–198.

Nikolaev, V.O., Bünemann, M., Schmitteckert, E., Lohse, M.J., and Engelhardt, S. (2006a). Cyclic AMP imaging in adult cardiac myocytes reveals far-reaching β1-adrenergic but locally confined β2-adrenergic receptor-mediated signaling. Circ. Res. *99*, 1084–1091.

Nikolaev, V.O., Gambaryan, S., and Lohse, M.J. (2006b). Fluorescent sensors for rapid monitoring of intracellular cGMP. Nat. Methods *3*, 23–25.

Nikolaev, V.O., Moshkov, A., Lyon, A.R., Miragoli, M., Novak, P., Paur, H., Lohse, M.J., Korchev, Y.E., Harding, S.E., and Gorelik, J. (2010). β2-adrenergic receptor redistribution in heart failure changes cAMP compartmentation. Science *327*, 1653–1657.

Paur, H., Wright, P.T., Sikkel, M.B., Tranter, M.H., Mansfield, C., O'Gara, P., Stuckey, D.J., Nikolaev, V.O., Diakonov, I., Pannell, L., et al. (2012). High levels of circulating epinephrine trigger apical cardiodepression in a β2-adrenergic receptor/Gi-dependent manner: a new model of Takotsubo cardiomyopathy. Circulation *126*, 697–706.

Razani, B., Woodman, S.E., and Lisanti, M.P. (2002). Caveolae: from cell biology to animal physiology. Pharmacol. Rev. *54*, 431–467.

Richter, W., Xie, M., Scheitrum, C., Krall, J., Movsesian, M.A., and Conti, M. (2011). Conserved expression and functions of PDE4 in rodent and human heart. Basic Res. Cardiol. *106*, 249–262.

Rona, G., Chappel, C.I., Balazs, T., and Gaudry, R. (1959). An infarct-like myocardial lesion and other toxic manifestations produced by isoproterenol in the rat. AMA Arch. Pathol. *67*, 443–455.

Rybin, V.O., Pak, E., Alcott, S., and Steinberg, S.F. (2003). Developmental changes in β2-adrenergic receptor signaling in ventricular myocytes: the role of Gi proteins and caveolae microdomains. Mol. Pharmacol. *63*, 1338–1348.

Shao, Y., Redfors, B., Ståhlman, M., Täng, M.S., Miljanovic, A., Möllmann, H., Troidl, C., Szardien, S., Hamm, C., Nef, H., et al. (2013a). A mouse model reveals an important role for catecholamine-induced lipotoxicity in the pathogenesis of stress-induced cardiomyopathy. Eur. J. Heart Fail. *15*, 9–22.

Shao, Y., Redfors, B., Scharin Täng, M., Möllmann, H., Troidl, C., Szardien, S., Hamm, C., Nef, H., Borén, J., and Omerovic, E. (2013b). Novel rat model reveals important roles of β-adrenoreceptors in stress-induced cardiomyopathy. Int. J. Cardiol. *168*, 1943–1950.

Shcherbakova, O.G., Hurt, C.M., Xiang, Y., Dell'Acqua, M.L., Zhang, Q., Tsien, R.W., and Kobilka, B.K. (2007). Organization of β-adrenoceptor signaling compartments by sympathetic innervation of cardiac myocytes. J. Cell Biol. *176*, 521–533.

Sohal, D.S., Nghiem, M., Crackower, M.A., Witt, S.A., Kimball, T.R., Tymitz, K.M., Penninger, J.M., and Molkentin, J.D. (2001). Temporally regulated and tissue-specific gene manipulations in the adult and embryonic heart using a tamoxifen-inducible Cre protein. Circ. Res. *89*, 20–25.

Stangherlin, A., Gesellchen, F., Zoccarato, A., Terrin, A., Fields, L.A., Berrera, M., Surdo, N.C., Craig, M.A., Smith, G., Hamilton, G., and Zaccolo, M. (2011). cGMP signals modulate cAMP levels in a compartment-specific manner to regulate catecholamine-dependent signaling in cardiac myocytes. Circ. Res. *108*, 929–939.

Stankovicova, T., Szilard, M., De Scheerder, I., and Sipido, K.R. (2000). M cells and transmural heterogeneity of action potential configuration in myocytes from the left ventricular wall of the pig heart. Cardiovasc. Res. *45*, 952–960.

Szentadrassy, N., Banyasz, T., Biro, T., Szabo, G., Toth, B.I., Magyar, J., Lazar, J., Varro, A., Kovacs, L., and Nanasi, P.P. (2005). Apico-basal inhomogeneity in distribution of ion channels in canine and human ventricular myocardium. Cardiovasc. Res. *65*, 851–860.

Thompson, W.J., and Appleman, M.M. (1971). Multiple cyclic nucleotide phosphodiesterase activities from rat brain. Biochemistry *10*, 311–316.

Usyk, T.P., Mazhari, R., and McCulloch, A.D. (2000). Effect of Laminar Orthotropic Myofiber Architecture on Regional Stress and Strain in the Canine Left Ventricle. J. Elast. Phys. Sci. Solids *61*, 143–164.

Wachten, S., Masada, N., Ayling, L.-J., Ciruela, A., Nikolaev, V.O., Lohse, M.J., and Cooper, D.M.F. (2010). Distinct pools of cAMP centre on different isoforms of adenylyl cyclase in pituitary-derived GH3B6 cells. J. Cell Sci. *123*, 95–106.

Willoughby, D., Wong, W., Schaack, J., Scott, J.D., and Cooper, D.M.F. (2006). An anchored PKA and PDE4 complex regulates subplasmalemmal cAMP dynamics. EMBO J. *25*, 2051–2061.

Wright, P.T., Nikolaev, V.O., O'Hara, T., Diakonov, I., Bhargava, A., Tokar, S., Schobesberger, S., Shevchuk, A.I., Sikkel, M.B., Wilkinson, R., et al. (2014). Caveolin-3 regulates compartmentation of cardiomyocyte beta2-adrenergic receptor-mediated cAMP signaling. J. Mol. Cell. Cardiol. *67*, 38–48.

Yang, J.H., Polanowska-Grabowska, R.K., Smith, J.S., Shields, C.W., 4th, and Saucerman, J.J. (2014). PKA catalytic subunit compartmentation regulates contractile and hypertrophic responses to β-adrenergic signaling. J. Mol. Cell. Cardiol. *66*, 83–93.

Cell Reports, Volume *23*

Supplemental Information

Cardiomyocyte Membrane Structure and cAMP Compartmentation Produce Anatomical Variation in β_2AR-cAMP Responsiveness in Murine Hearts

Peter T. Wright, Navneet K. Bhogal, Ivan Diakonov, Laura M.K. Pannell, Ruwan K. Perera, Nadja I. Bork, Sophie Schobesberger, Carla Lucarelli, Giuseppe Faggian, Anita Alvarez-Laviada, Manuela Zaccolo, Timothy J. Kamp, Ravi C. Balijepalli, Alexander R. Lyon, Sian E. Harding, Viacheslav O. Nikolaev, and Julia Gorelik

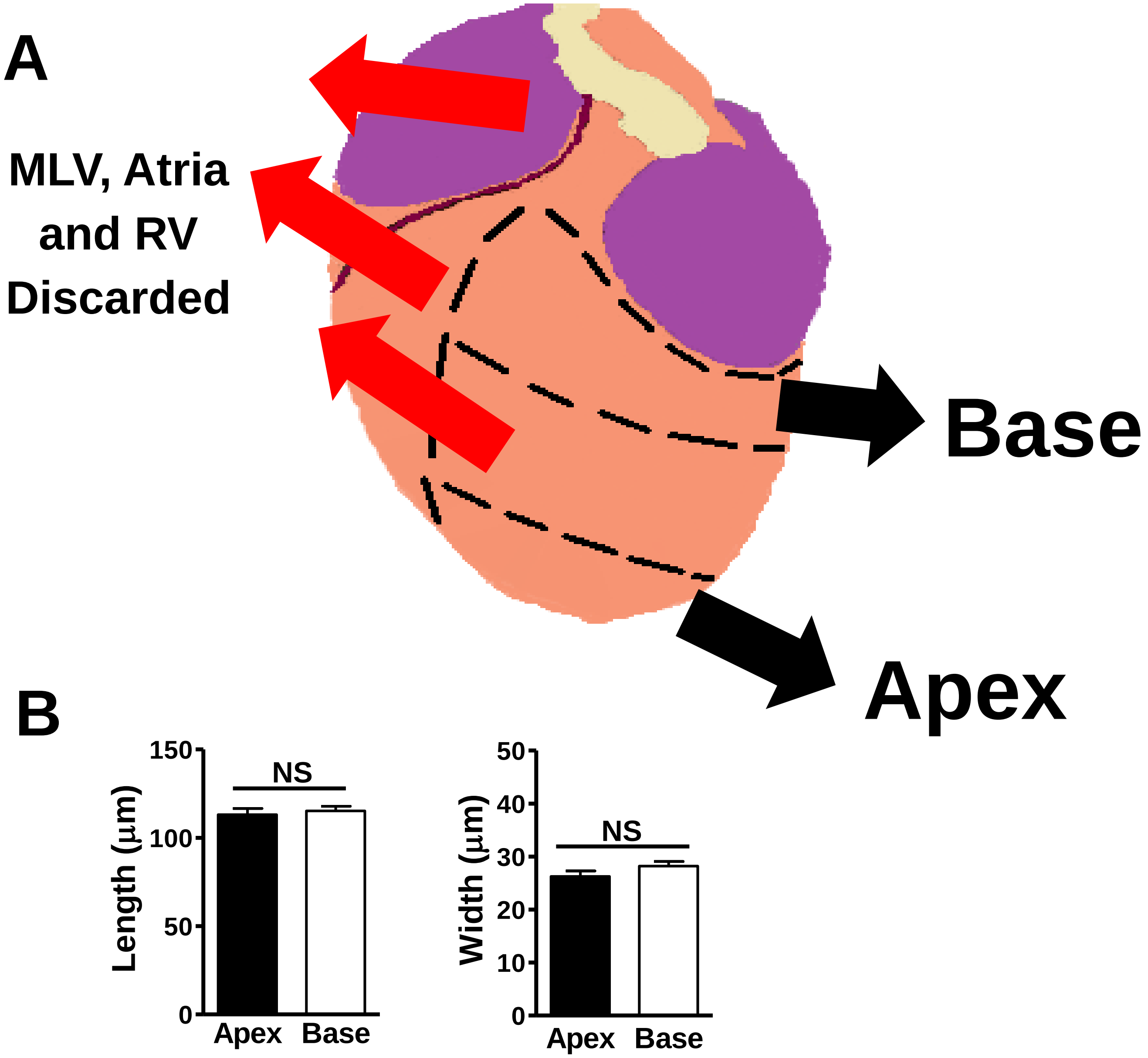

Figure S1. Diagram demonstrating the isolation of cardiomyocytes from separate myocardial regions. Related to Figure 1.

a) Following an initial perfusion step with enzymes introduced via Langendorff perfusion, the mid-left ventricle (MLV), atria and right ventricle (RV) are discarded. The basal and apical sections of the left ventricle are then disaggregated in separate vessels to provide pure populations of apical or basal cardiomyocytes. **b)** Cell length and width of apical and basal cardiomyocytes from rat (µm) N,n (3)50.

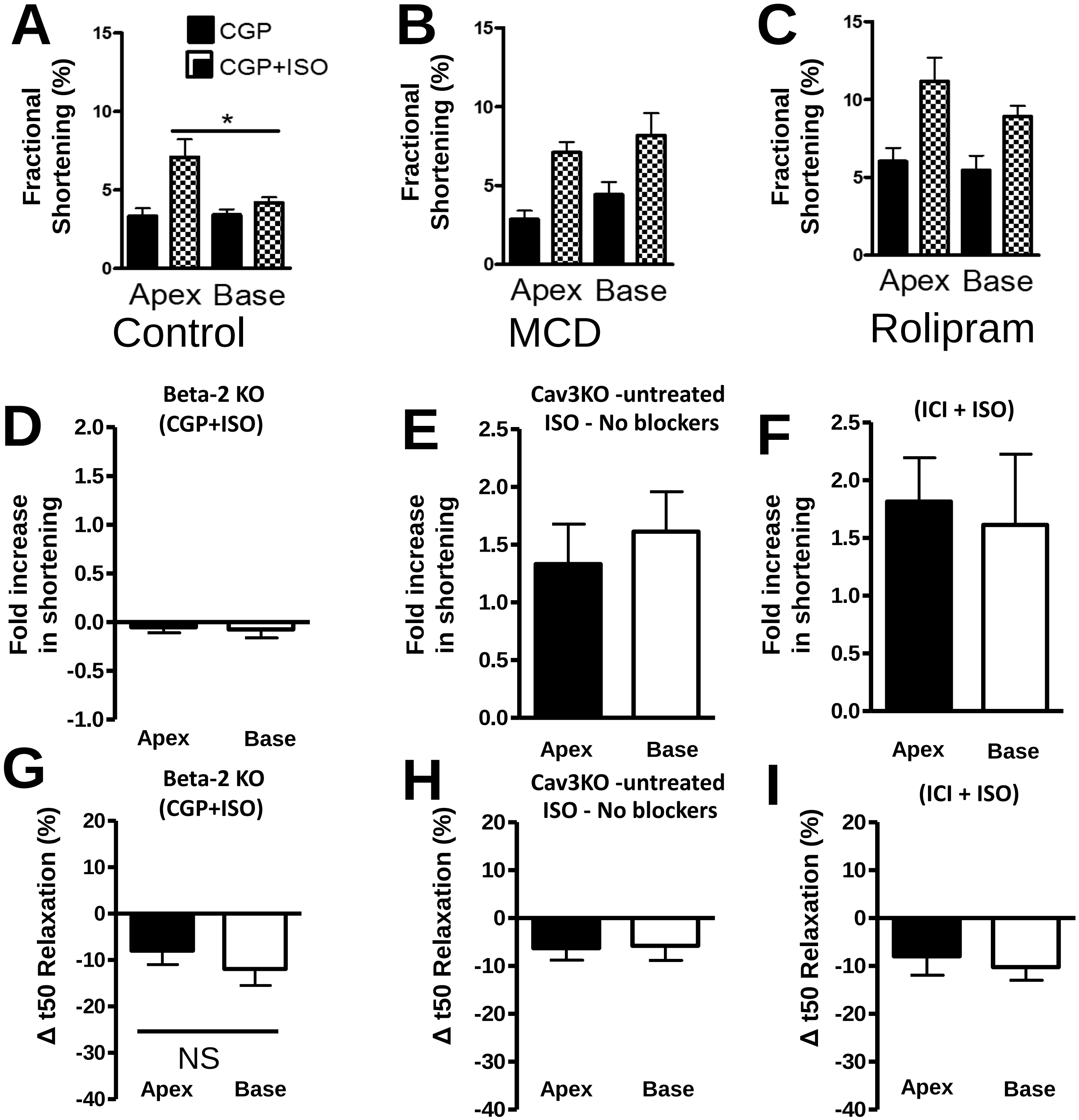

Figure S2 Contraction of wild type rat myocytes from apex and base, before and after stimulation of β_2AR. Contraction of β_2AR KO and Cav3KO (untreated) myocytes. Related to Figure 1, 3 and 5.
a. Untreated cells N,n=(4)11/9; **b.** cells treated with methyl-ß-cyclodextrin N,n=(4)6/9; **c.** or rolipram N,n=(3)6/6. **d**) Fractional shortening response of β_2AR KO cardiomyocytes CGP+ISO stimulation N, n=(4) 9. **e**) Fractional shortening response of Cav3KO cardiomyocytes following ISO stimulation. N,n=(4) 8-11. **f**) Fractional shortening response of Cav3KO cardiomyocytes following β_1AR stimulation (ICI118, 551 (β_2AR blocker) + ISO). N,n=(4) 8 **g**) Change in relaxation response. N,n=(4) 8-11. **h**) Change in relaxation response (ICI118, 551 (β_2AR) + ISO). N,n=(4) 8 h)). **i**) Change in relaxation response N, n= (4) 9.

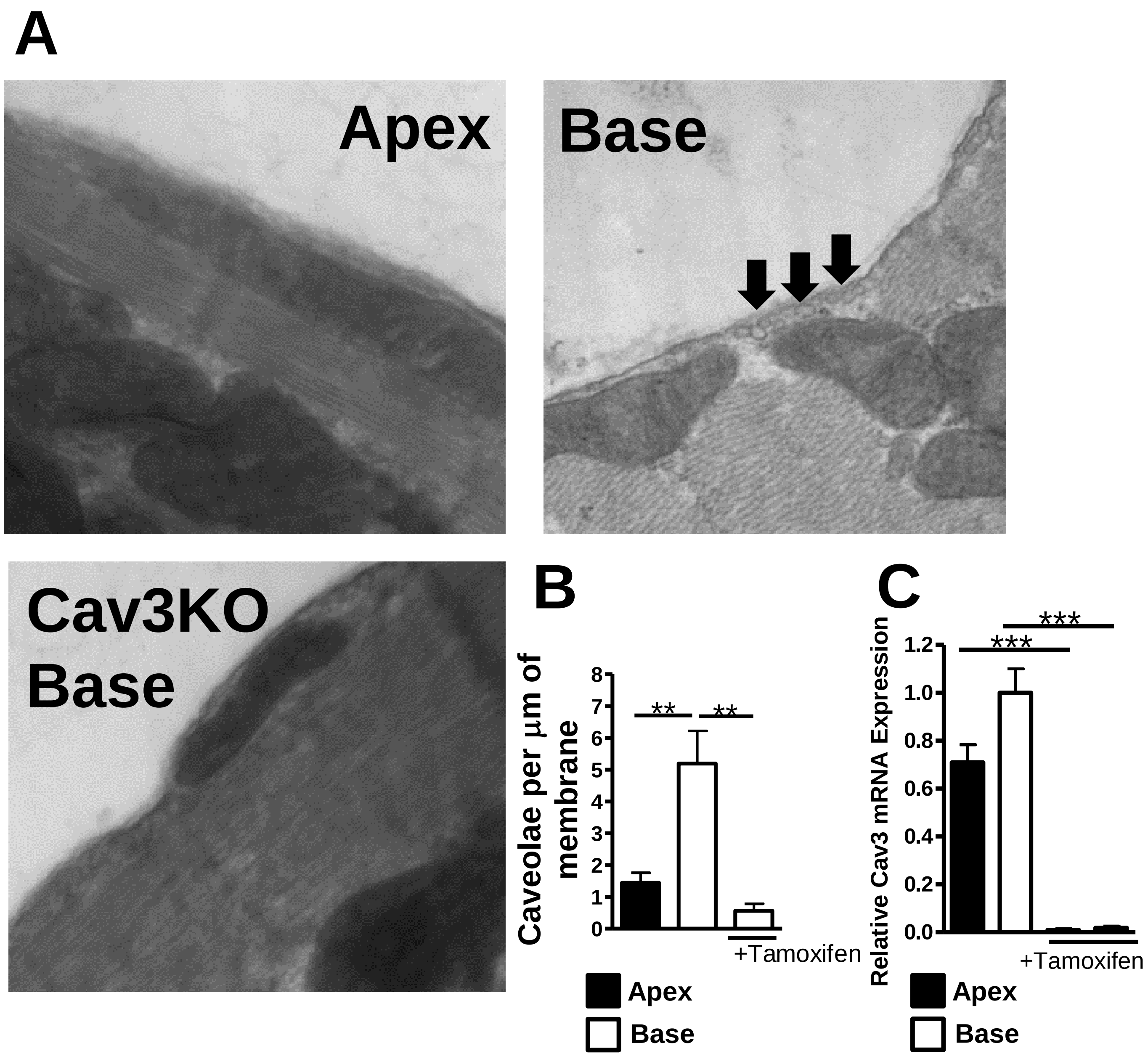

Figure S3. Characteristics of the Cav-3 KO mouse. Related to Figure 3.
a) Representative electron micrographs of normal apical and basal mouse cardiac myocytes and a basal ventricular myocyte isolated from a Cav3KO mouse. **b**) Quantification of the average amount of caveolae per µm of membrane length (N=10 frames/10 cells; $p<0.001$) in Cav3KO mice with or without tamoxifen administration **c)** Quantification of the relative expression of Cav3 mRNA in apical and basal myocytes of Cav3KO mice with or without tamoxifen treatment.

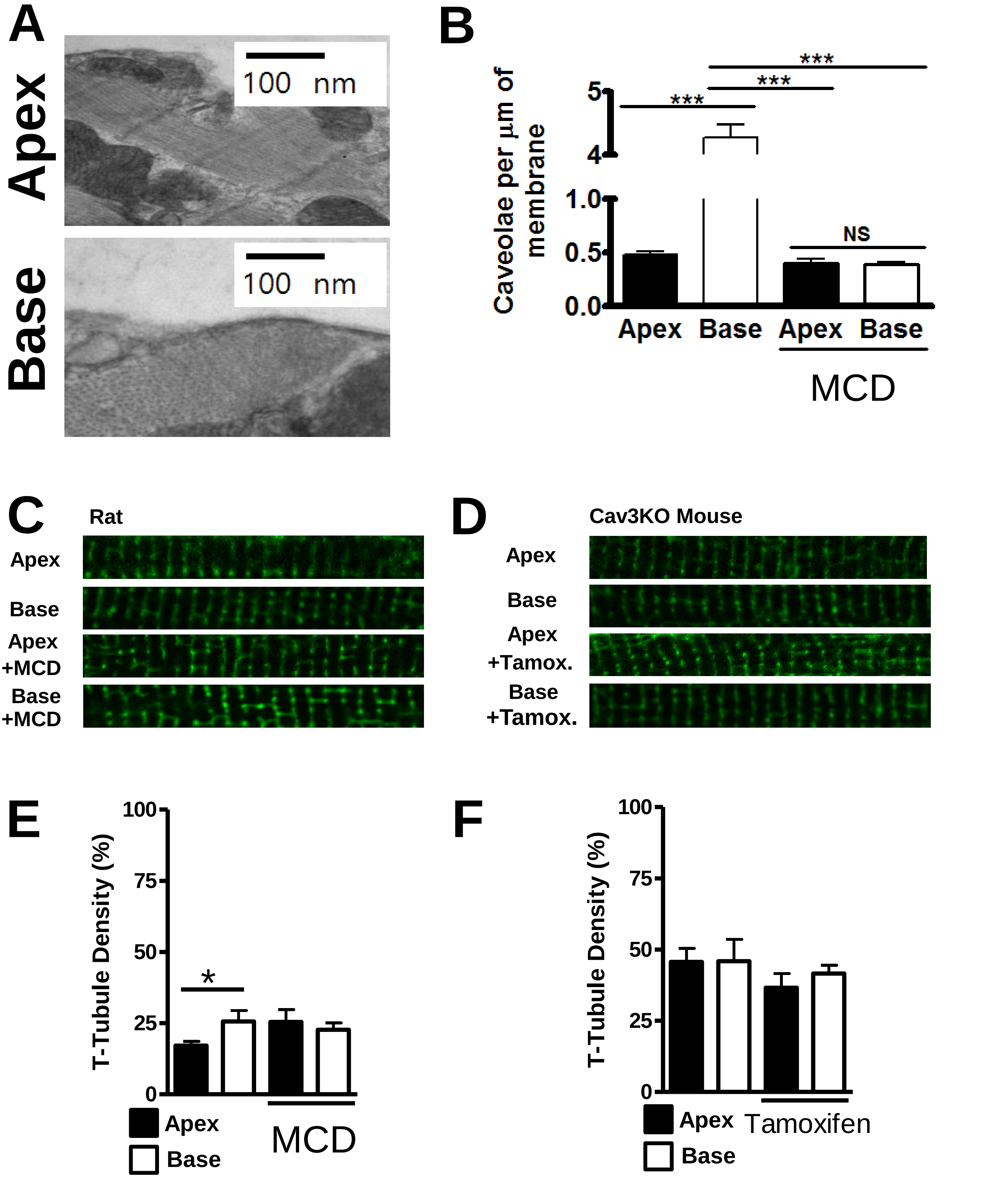

Figure S4. The removal of caveolae with MCD and validation of t-tubular density following cell treatment. Related to Figure 3 and 4.
a) Representative electron micrographs of rat apical and basal cardiomyocytes following treatment with methyl-β cyclodextrin **b)**. This leads to the removal of caveolae. **c)** Representative images of apical and basal cells from rat stained with di-8ANNEPPS with or without MCD pre-treatment. **d)** Representative images of apical and basal cells from Cav3KO mice stained with di-8ANNEPPS with or without tamoxifen induced KO of caveolin-3. **e,f)** quantification of t-tubule density.

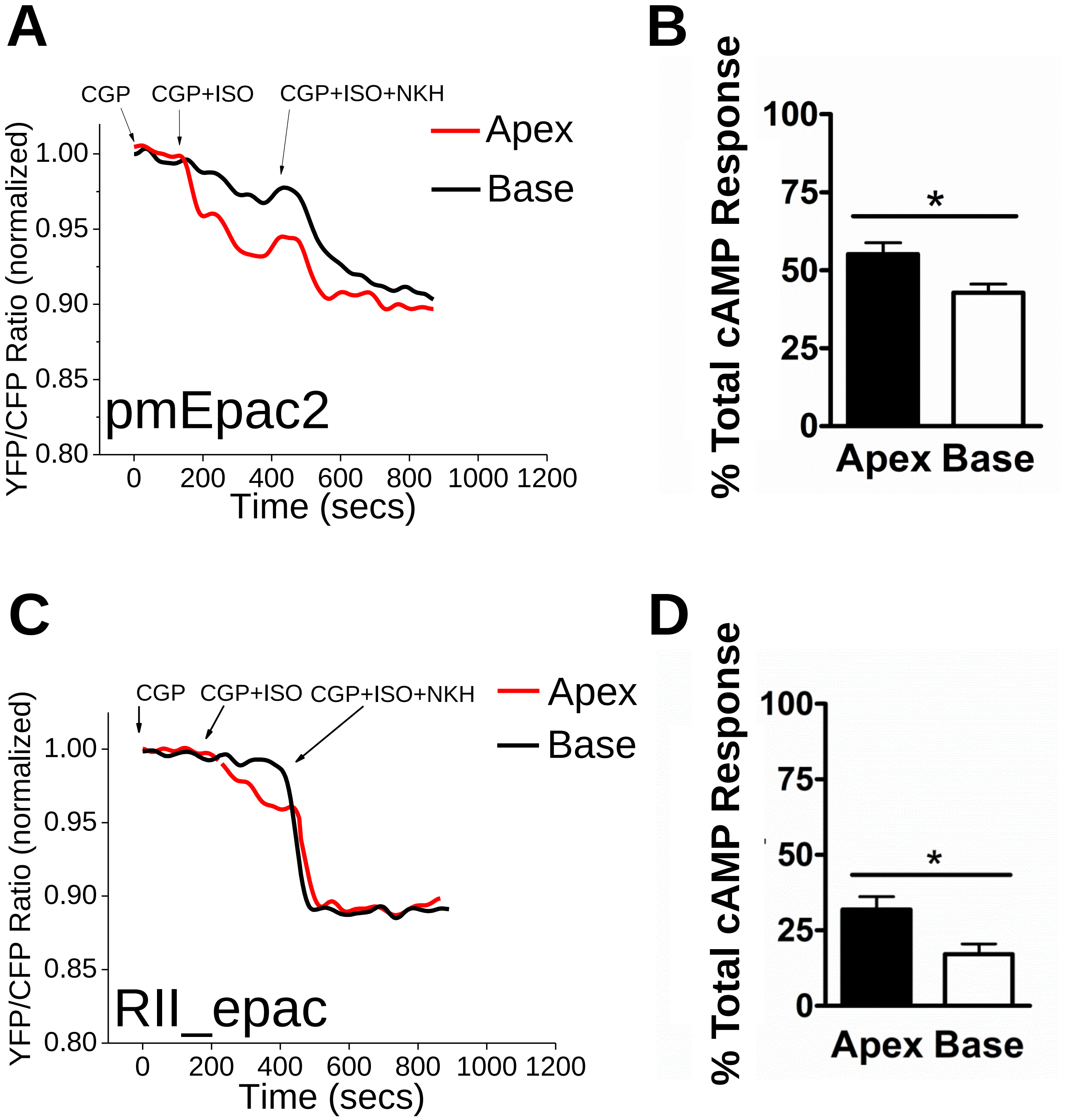

Figure S5. Stimulated production of cAMP in plasma membrane or RII_Epac nanodomains. Related to Figure 4. **a)** Representative traces of FRET responses in apical and basal cardiomyocytes from rat as measured by the pmEPAC2 sensor,in the plasma membrane/caveolar nanodomains (**a**, **b**) N,n=(5)21/24, in the PKA_RII nanodomains. (**c**, **d**). N,n=(4)13/13.

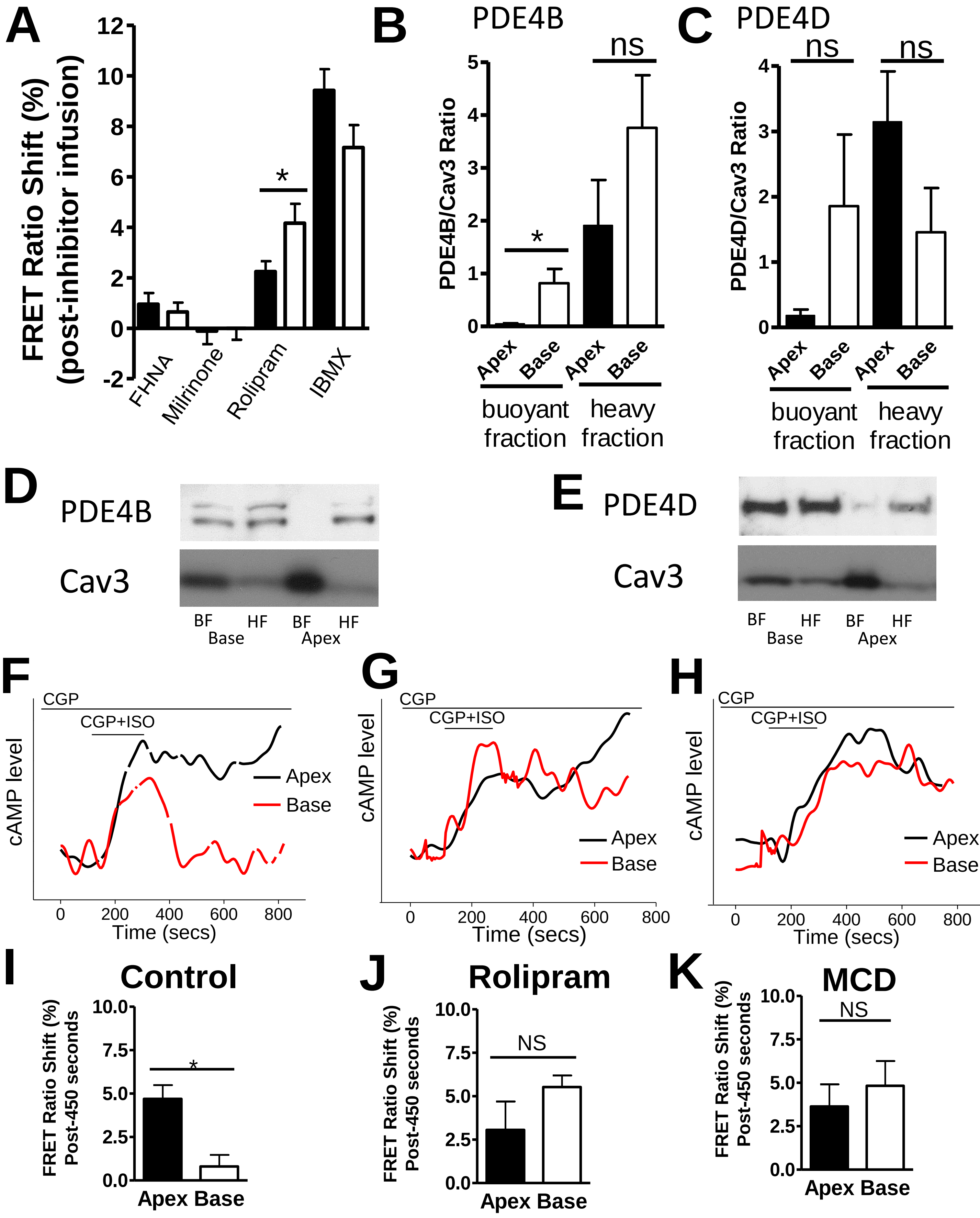

Figure S6. Increased levels of PDE4 and PDE4 activity are present in apical cardiomyocytes. Related to Figure 5.
a) FRET responses in cEPAC2 transfected cells following β_2AR stimulation and perfusion with inhibitors of PDE2 (EHNA) N,n=(4)14/14, PDE3 (milrinone 1 μM) N,n=(4)12/14, PDE4 (rolipram 10 μM) N,n=(5)21/24 or general PDE inhibitor (IBMX 100 μM) N,n=(4)12/14. **b, d)** Quantification of PDE4B corrected to caveolin-3 levels (N=3, 4). **c, e)** Quantification of PDE4D corrected to caveolin-3 levels (N=3, 4). The persistence of cAMP in RII compartments of apical and basal myocytes from rat. **f, g, h.** Representative traces of washout experiments with control myocytes from apex and base N,n=(3) 6,4, or cells treated with rolipram N,n=(3) 6,10 and methyl-ß-cyclodextrin N,n=(3) 4,4 ; **i, j, k**. quantification of washout data at 450 seconds from the start of experiment.

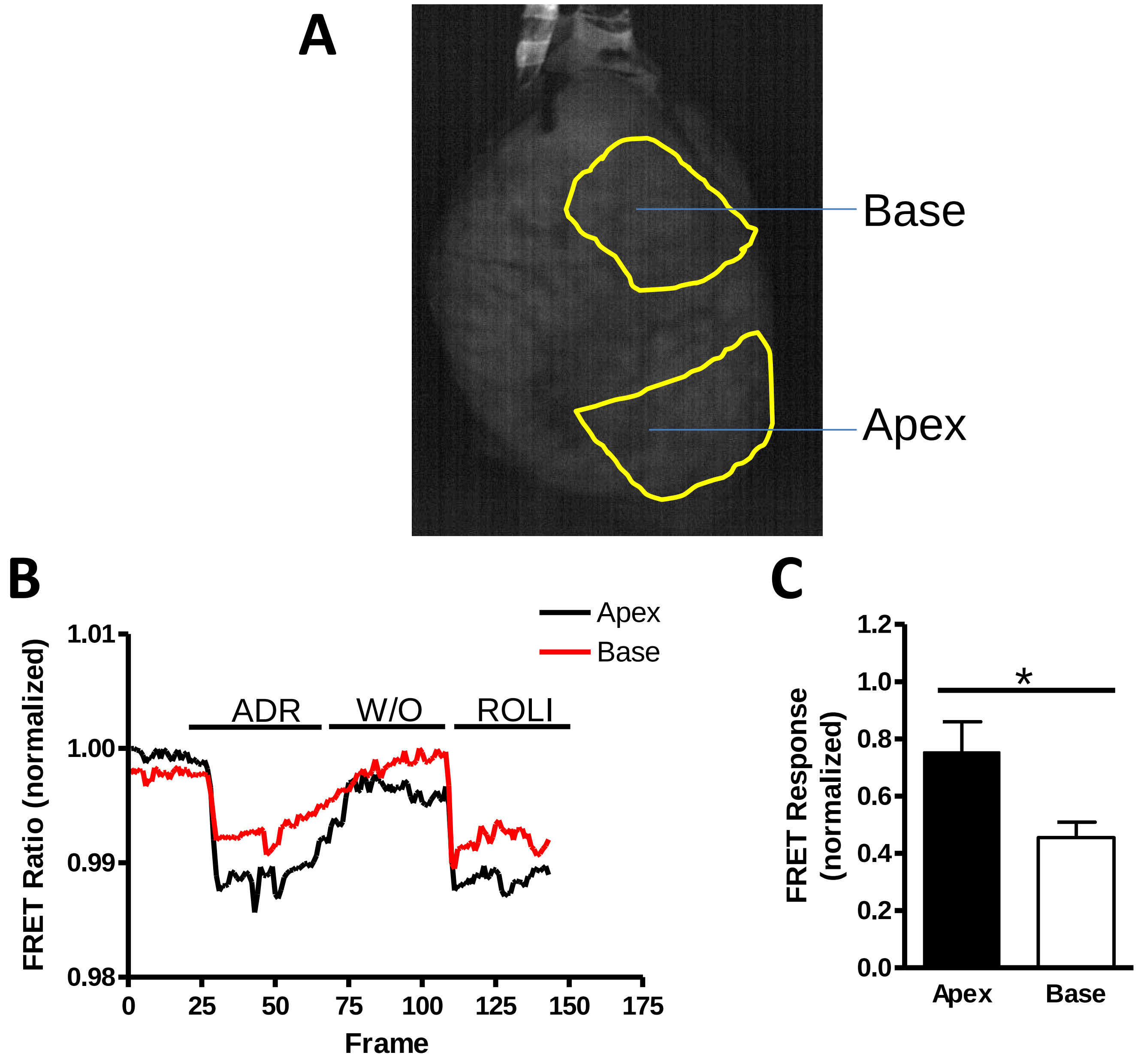

Figure S7. Regional response of pmEpac1 FRET sensor to epinephrine stimulation. Related to Figure 6.
a) Fluorescent image of the entire heart preparation from the pmEpac1 mouse with apical and basal regions indicated. **b)** Time trace showing the response of the two myocardial regions following infusion of epinephrine (ADR, 100nM), wash out (W/O) and subsequent application of rolipram (ROLI, 10 µM). **c)** Quantification of the average responses of apical and basal regions during epinephrine stimulation phase (N=4).

Supplementary Methods

RNA Isolation and Reverse-Transcription Quantitative Polymerase Chain Reaction

Flash frozen tissue from apex or base of WT and Cav3 KO mice were thawed. RNA was isolated using the RNeasy Mini Kit (Qiagen). Complementary DNA (cDNA) was made from 500 ng of isolated RNA using the iScript RT Supermix (Bio-Rad) for reverse transcription. Cav3 mRNA quantification was performed using Taqman Universal PCR Master Mix (Thermo Fisher Scientific) and Taqman Gene Expression Assays for Cav3 (Applied Biosystems, probe Mm01182632_m1) and Gapdh (IDT, probe Mm.PT.39a.1). The expression of Cav3 was normalized to the endogenous control, Gapdh to calculate ΔCq with Cq as the quantification cycle. The relative expression of Cav3 for each group was calculated as 2-ΔCq and normalized to Cav3 expression in the WT base.

Cell fractionation and Western blotting to establish PDE4 subtype expression

Freshly isolated apical and basal cardiomyocytes were and subsequently fractionated in a sucrose-density column by ultracentrifugation as per the method of Head et al. (See PDE assay section). Buoyant and heavy fractions were retained. These samples were then lysed in 10mM HEPES buffer containing 300mM sucrose, 150mM NaCl, 1mM EGTA, 2mM CaCl2 and 1 % Triton-X and homogenized by pulling the lysate 5x through an insulin cannula (BRAUN, U-40 Insulin, 30Gx1/2, #4001525) followed by 15 min incubation on ice. After centrifugation of the lysate for 5 min at 8000 rpm and 4°C, the supernatant was separated from the pellet and used for protein concentration measurements using a PierceTM BCA Protein Assay kit. Lysates were enriched with 3x reducing buffer (3:1) and 10ug of protein/well were loaded and run on 10% SDS-PAGE gels. For immunoblotting rabbit polyclonal antibodies against PDE4B and 4D were used (1:1000), as well as mouse-monoclonal anti-caveolin-3 (Cav-3) (Applied Biosystems, UK). The optical density of bands were analysed using the freeware ImageJ (available at: https://imagej.nih.gov/ij/).

Determination of membrane regularity and density

Di-8-ANEPPS was used to stain the membranes of isolated cardiomyocytes on day of isolation. These were imaged with an inverted confocal microscope (Zeiss LSM-780). Confocal slices of the cells were generated; T-tubule regularity and density were quantified from 2D images using ImageJ and a bespoke MatLab script (Ibrahim et al., 2010).

Determination of regularity and density of caveolin-3 containing structures

Cardiomyocytes were fixed with 4% paraformaldehyde on day of isolation. Cell membranes were blocked with 10% FBS and immuno-labelled with mouse-monoclonal anti-caveolin-3 (Cav-3) (Applied Biosystems, UK). Alexa-488 conjugated anti-IgG (donkey anti-mouse) was used to provide secondary labelling. Cells were analysed by confocal microscopy.

Transmission Electron Microscopy

Apical and basal cardiomyocytes were fixed in glutaraldehyde, washed incorporated into agar and then embedded in araldite. Ultrathin sections 0.1µm thick were stained with uranyl acetate and lead citrate. Images taken with a transmission electron microscope were then used to assess the number of caveolar nanodomains present in cellular membranes. Ten representative fields of view were acquired for each cell and both open and closed caveolae were counted.

Phosphodiesterase activity assay

Apical and basal cardiomyocytes were plated on 100mm Laminin coated petri dishes and allowed to attach for an hour and treated as per the method of Head et al. (Head et al., 2005), to allow the preparation of caveolar and non-caveolar membrane fractions. Cells were scraped into a buffer containing Na2CO3 at pH 11.0 The cells were homogenized and the resulting suspension mixed with a sucrose-containing Na2CO3 buffer to create a 45% sucrose buffer. This was transferred to an ultracentrifuge tube and further layers of lower density sucrose buffers (35% and 5%) were applied. These manipulations were performed at 4oC and in the presence of phosphatase and protease inhibitors (Roche, DE). Samples were centrifuged for 16hrs at 4oC and 200,000g. Caveolar and non-caveolar membrane fractions were identified (Head et al., 2005) these preparations were isolated by sequentially aspirating segments of the sucrose gradient. Samples were stored at -80oC until use. PDE assay was an adaptation of a previous method (Appleman and Thompson, 1971) to determine the cAMP hydrolysis capacity of tissue preparations. The caveolar and non-caveolar samples from apical and basal cardiomyocytes were used for a PDE activity assay. Samples were exposed to Tritiated 3’-5’ cAMP with or without 10µM rolipram (PDE4 specific inhibitor) or 100 µM IBMX (non-specific inhibitor) for 60 minutes. The assay was terminated via boiling the mixture for 1 minute. After a brief cool-down period, the mixture was exposed to snake venom nucleotidase for 25 minutes at 30oC. The reaction mix was then loaded on self-made flow-through columns containing 100mg of Bio-Rad resin, AG1-X2, 200-400 mesh. The amount of 3H-Adenosine left in the eluent was then quantified by liquid scintillation as a measure of the cAMP hydrolysis capacity of the preparation and the relative ability of PDE blockade to interfere with this.
